# Recruitment of α4β7 monocytes and neutrophils to the brain in experimental colitis is associated with elevated cytokines and anxiety-like behavior

**DOI:** 10.1186/s12974-022-02431-z

**Published:** 2022-04-04

**Authors:** Nina L. Cluny, Kewir D. Nyuyki, Wagdi Almishri, Lateece Griffin, Benjamin H. Lee, Simon A. Hirota, Quentin J. Pittman, Mark G. Swain, Keith A. Sharkey

**Affiliations:** 1 Hotchkiss Brain Institute, Cumming School of Medicine, University of Calgary, Calgary, AB Canada; 2 Snyder Institute for Chronic Diseases, Cumming School of Medicine, University of Calgary, Calgary, AB Canada; 3 Department of Physiology and Pharmacology, Cumming School of Medicine, University of Calgary, 3330 Hospital Drive NW, Calgary, AB T2N 4N1 Canada; 4 Division of Gastroenterology and Hepatology, Department of Medicine, Cumming School of Medicine, University of Calgary, Calgary, AB Canada; 5 Alberta Children’s Hospital Research Institute, Cumming School of Medicine, University of Calgary, Calgary, AB Canada

**Keywords:** MAdCAM-1, Neutrophil, Monocyte, Colitis, Interleukin 1β, Anxiety-like behavior

## Abstract

**Background:**

Behavioral comorbidities, such as anxiety and depression, are a prominent feature of IBD. The signals from the inflamed gut that cause changes in the brain leading to these behavioral comorbidities remain to be fully elucidated. We tested the hypothesis that enhanced leukocyte–cerebral endothelial cell interactions occur in the brain in experimental colitis, mediated by α4β7 integrin, to initiate neuroimmune activation and anxiety-like behavior.

**Methods:**

Female mice treated with dextran sodium sulfate were studied at the peak of acute colitis. Circulating leukocyte populations were determined using flow cytometry. Leukocyte–cerebral endothelial cell interactions were examined using intravital microscopy in mice treated with anti-integrin antibodies. Brain cytokine and chemokines were assessed using a multiplex assay in animals treated with anti-α4β7 integrin. Anxiety-like behavior was assessed using an elevated plus maze in animals after treatment with an intracerebroventricular injection of interleukin 1 receptor antagonist.

**Results:**

The proportion of classical monocytes expressing α4β7 integrin was increased in peripheral blood of mice with colitis. An increase in the number of rolling and adherent leukocytes on cerebral endothelial cells was observed, the majority of which were neutrophils. Treatment with anti-α4β7 integrin significantly reduced the number of rolling leukocytes. After anti-Ly6C treatment to deplete monocytes, the number of rolling and adhering neutrophils was significantly reduced in mice with colitis. Interleukin-1β and CCL2 levels were elevated in the brain and treatment with anti-α4β7 significantly reduced them. Enhanced anxiety-like behavior in mice with colitis was reversed by treatment with interleukin 1 receptor antagonist.

**Conclusions:**

In experimental colitis, α4β7 integrin-expressing monocytes direct the recruitment of neutrophils to the cerebral vasculature, leading to elevated cytokine levels. Increased interleukin-1β mediates anxiety-like behavior.

**Supplementary Information:**

The online version contains supplementary material available at 10.1186/s12974-022-02431-z.

## Introduction

Inflammatory bowel diseases (IBD) are chronic inflammatory conditions of the gastrointestinal (GI) tract with a relapsing and remitting time course and a multifactorial (i.e., genetic, environmental and immune) etiology [1, 2]. Patients experience abdominal pain, diarrhea, rectal bleeding and weight loss during disease exacerbations, and may continue to experience pain and altered gut function, even when inflammation has resolved [3–5]. Behavioral comorbidities are a prominent feature of IBD. Individuals with IBD experience depression, anxiety, fatigue, decreased sociability, sleep disturbances and cognitive dysfunction [6–10]. These cognitive, emotional and behavioral abnormalities occur more commonly in women. They are observed during active disease and when it is in remission and have a significant negative impact on quality of life. Despite significant advances in understanding the pathogenesis of IBD, the signals from the inflamed gut that bring about changes in the brain that lead to behavioral comorbidities remain to be fully elucidated.

The central nervous system (CNS) senses and integrates signals originating from the GI tract, providing a dynamic imprint of the state of the gut [11, 12]. Signaling from the gut to the brain is complex, and involves neural, humoral, microbial and cellular mediators [13, 14]. In addition to peripheral neural pathways and circulating factors that directly and indirectly access the CNS, it is becoming increasingly clear that the immune system communicates with the brain [14–16]. When inflammation occurs in the body, activated immune cells produce mediators, including cytokines, that communicate changes in peripheral immunity to the CNS via all these pathways. The cellular pathways include the direct trafficking of activated immune cells to the brain, which then sets in motion neuroimmune and cellular mechanisms that ultimately alter excitability of the CNS, leading to behavioral changes [14, 16–18]. We and others have documented that animal models of IBD recapitulate many of the behavioral changes observed in patients, including anxiety-like and depressive-like behaviors [19–25]. Altered neural and immune signaling in the CNS have also been demonstrated in these animal models of IBD [17, 20, 26–28].

The molecular signaling mechanisms that guide trafficking of activated immune cells to the brain in IBD and experimental colitis are not well understood. In contrast, trafficking of immune cells to the inflamed gut in IBD has received significant experimental and clinical attention as a therapeutic target to decrease gut inflammation [29–31]. Specifically, the integrin, α4β7 was found to be a key regulator for intestinal homing of lymphocytes through binding to mucosal addressin cell adhesion molecule-1 (MAdCAM-1), which is upregulated in response to intestinal inflammation [32, 33]. This important observation led to the development and clinical use of the α4β7 monoclonal antibody vedolizumab for treating IBD [34, 35]. However, it has become clear that α4β7 is expressed not only on gut-homing lymphocytes, but also on monocyte subsets that can regulate intestinal inflammation [36, 37]. The potential role of α4β7 integrin-expressing leukocytes in immune cell trafficking to the brain remains to be shown, but it is noteworthy that IBD patients treated with vedolizumab not only have improved colitis, but also an improvement in sleep quality and mood [38], suggesting that α4β7 integrin inhibition can regulate behavior, possibly by altering immune cell trafficking to the brain.

We investigated immune cell trafficking in the well-characterized animal model of IBD, dextran sodium sulphate (DSS)-induced colitis[39]. We tested the hypothesis that enhanced leukocyte–cerebral endothelial cell interactions occur in the brain in experimental colitis, mediated by α4β7 integrin, to initiate neuroimmune activation and anxiety-like behavior. We show that α4β7 integrin-expressing monocytes within the circulation direct the recruitment of neutrophils to the cerebral vasculature, leading to elevated levels of the proinflammatory cytokines interleukin (IL)-1β and CCL2. Elevated IL-1β mediates anxiety-like behavior in this model of experimental colitis.

## Materials and methods

### Animals

Female (16–22 g) and male (22–30 g) C57BL/6 J mice (The Jackson Laboratory, Bar Harbor, ME, USA) aged 7–8 weeks on arrival, were group housed (3–4 mice per cage), under a 12 h light–dark cycle (lights off 19:00) in plastic sawdust floor cages (22°C, 40% humidity, standard laboratory chow and water ad libitum) in a specific pathogen-free facility. After one week of acclimatization, cages of mice were randomly assigned to treatment groups. All experimental procedures were approved by the Health Sciences Animal Care Committee of the University of Calgary and were carried out in accordance with the guidelines of the Canadian Council on Animal Care (Animal Use Protocols AC17-0093, AC15-0129). In the majority of studies, female mice were used, since IBD comorbidities more commonly occur in females [6–10]. To confirm some of our main findings we also employed male mice, as described below.

### DSS-induced colitis

Mice were given DSS (Catalog #14489, 40–50 kDa, Affymetrix, Cleveland, OH, USA) ad libitum in their drinking water (2.5–3.5% wt/vol); on day 5 this was replaced with tap water until day 7. Control mice received tap water alone for 7 days. For these studies, we used various different batches of DSS. Since the efficacy of DSS varies by batch, we ran pilot studies for the DSS damage and then chose the concentration that gave us equivalent colonic damage scores, ensuring to the extent possible we would have a similar degree of colitis in all our experiments. Body weight was measured three times per week. Day 7 has previously been determined to be the peak of colonic inflammation in this model [19].

In all cases, mice were euthanized by cervical dislocation under isoflurane or ketamine–xylazine anesthesia on day 7. Body weight score was calculated as the % weight loss on day 7 from the initial body weight on day 0 (0 = 0%, 1 =  < 0– ≤ 5%, 2 =  > 5– ≤ 10%, 3 =  > 10– ≤ 15%, 4 =  > 15%). The colon was dissected and examined by a blinded observer for macroscopic evidence of colitis. Colon length score was calculated as a % of control colon length, with the average control length in females being 6.0 cm and in males 7.4 cm: (0 = 85–100%, 1 = 75–84%, 2 = 65–74% and 3 < 65%). The presence (score = 1) or absence (score = 0) of adhesion, erythema, gross fecal blood and diarrhea was recorded. A total damage score was calculated for each animal comprising, body weight score, adhesion, colon length score, erythema score, fecal blood score, diarrhea score, length of inflamed colon as % of total length, ulcer length and bowel thickness (mm). The macroscopic damage score is presented as mean ± standard error of the mean (SEM) for ease of comparison with the literature.

### Flow cytometry

To determine the proportions of leukocyte population in colitis, we performed a flow cytometric analysis. After cervical dislocation, blood was immediately withdrawn by cardiac puncture. A whole blood staining method was used to investigate the phenotypic profile of peripheral blood leucocytes. In order to block non-specific binding to Fc III/II receptors, 100 µL of anticoagulated whole blood were added to a 5-mL polystyrene tube and incubated with anti-CD16/CD32. Following incubation at room temperature for 15 min, a predetermined optimum concentration of desired fluorochrome-conjugated primary antibodies was added and incubated for 30 min. Red blood cells were lysed by adding 2 mL of ammonium-chloride-potassium lysis buffer. Following incubation at room temperature for a further 10 min, cells were washed twice in staining buffer by centrifugation at 500×*g* for 10 min. Samples were acquired either using a FACScan flow cytometer (Becton Dickinson, Mountain View, CA, USA) or Attune™ Acoustic Focusing flow cytometer (Applied Biosytems, Mainway, Burlington, ON, Canada). Data were analyzed using FlowJo® software (Treestar, Ashland, OR, USA). Flow cytometry dot-plots showing the gating strategy used in the identification of α4β7 expressing monocytes and neutrophils in mouse blood are shown in Additional file 1: Fig. S1. The following antibodies were obtained from sources indicated: anti-mouse CD16/CD32 (93), eBioscience™ catalog # 14-0161-82; anti-mouse Ly6C (HK1.4), PerCP-Cyanine5.5, eBioscience™Catalog # 45-5932-82, ThermoFisher Scientific, Waltham, MA, USA. Anti-mouse CD11b (M1/70), Alexa Fluor-700, BioLegend catalog # 101222; anti-mouse Ly6G (1A8) PE/Cyanine7, BioLegend catalog # 127618; anti-mouse CD3ε (145-2C11), Brilliant Violet 510, BioLegend catalog # 100353; anti-mouse Integrin α4β7 (DATK32), APC BioLegend catalog # 120608; BioLegend, San Diego, CA, USA. Data are shown as mean ± SEM of 5 mice per group. For comparisons between two groups, an unpaired Student's *t*-test was performed (GraphPad Prism version 9, GraphPad, San Diego, CA, USA). A P value of ≤ 0.05 was considered significant.

### Intravital microscopy

To examine leukocyte–endothelial interactions in colitis, we performed intravital microscopy using previously published approaches [20, 40]. On day 7 of colitis, mice were anesthetized using a ketamine and xylazine mixture (Intraperitoneal (IP).; 200 mg/kg and 10 mg/kg, respectively). The tail vein was cannulated for administration of dyes and conjugated antibodies for imaging. The skin was blunt dissected from the skull and the parietal bone thinned to approximately 30 μm using a high-speed dental drill, resulting in an intact cranial window over the parietal cortex of approximately 5 mm × 5 mm [41]. The window was covered with a drop of saline and the mouse placed on the microscope stage.

Dyes or conjugated antibodies were administered intravenously immediately before imaging: Rhodamine-6G (0.225 mg/kg; catalog #252433, Sigma-Aldrich, St. Louis, MO, USA) was used to visualize all leukocytes [17], phycoerythrin (PE)- or allophycocyanin (APC)-labeled CD-31 (390; 2 μg/mouse; eBioscience; catalog #17-0311-82 (APC)- #12-0311-81, (PE)) was used to label cerebral endothelial cells, PE-labeled Ly6G (1A8, 2 μg/mouse; eBioscience; catalog #12-9668-80) was used to label neutrophils[17], APC-labeled Ly6C (HK1.4; 2 μg/mouse; eBioscience; catalog #17-5932-80) was used to label monocytes, and APC-labeled MAdCAM-1 (MECA-367; 2 μg/mouse; Biolegend, San Diego, CA, USA; catalog #120711) was used to label MAdCAM-1.

For the depletion of specific cells, mice with colitis were IP administered antibodies or isotype control, 18–22 h prior to the imaging experiment. Circulating neutrophils were depleted using anti-Ly6G (1A8; 200 μg/mouse; Bio X Cell, Lebanon, NH, USA; catalog #BE0075-1), while the controls received rat IgG2a antibody (200 μg/mouse; Bio X Cell; catalog #BE0089), as previously reported [42–44]. The efficiency of the mAb anti-Ly6G to specifically deplete classical monocytes in C57BL/6 mice was confirmed using flow cytometric analysis as shown in Additional file 1: Fig. S2. Anti-α4 integrin (PS/2; 200 μg/mouse; Bio X Cell; catalog #BE0071) and anti-α4β7 integrin (DATK32; 200 μg/mouse; Bio X Cell; catalog #BE0034) were administered to investigate the role of integrins in leukocyte recruitment. The controls were administered rat IgG2b (200 μg; Bio X Cell; catalog #BE0090) and rat IgG2a, respectively. Other mice were administered anti-MAdCAM-1 (MECA-367; 200 μg/mouse; Bio X Cell; catalog #BE0035) or rat IgG2a. Circulating monocytes were depleted using anti-Ly6c (HK1.4; 100 μg/mouse; eBioscience; catalog #16-5932-85), with controls receiving rat IgG2c (100 μg; Biolegend; catalog #400710). The efficiency of the mAb anti-Ly6C to specifically deplete classical monocytes in C57BL/6 mice was confirmed using flow cytometric analysis as shown in Additional file 1: Fig. S3. We recognize that it would be optimal to use a fluorescently conjugated, non-competing clone targeting the same cellular antigen to evaluate the efficacy of cell depletion by FACS, but the lack of availability of rigorously validated antibodies to do this limits this approach. The monocyte-specific antibody clone HK1.4 was used to deplete monocytes in vivo, and also to evaluate the efficacy of cell depletion by FACS. When examining blood samples from HK1.4-treated mice, a Ly6C^low^ cell population is readily detectable by flow cytometry indicating that the antibody used for in vivo cell depletion does not fully occupy all binding sites for the HK1.4 specific epitope (Additional file 1: Fig. S3). The Ly6C^hi^ cell population would be expected to be readily detected by flow cytometry analysis given that this cell population, by definition, has a higher expression of the antigen than the LY6C^low^ population. We have previously used this anti-alpha4 integrin neutralizing antibody strategy to prevent monocyte adhesion to cerebral endothelial cells [45].

Leukocyte–endothelial interactions were imaged in pial vessels of the meninges of the parietal cortex using a Quorum WaveFX spinning disk confocal microscope (Quorum Technologies, Puslinch, ON, Canada) driven by Volocity 6.1 acquisition software (PerkinElmer, Waltham, MA, USA). Labeled cells were imaged using 561, or 635 nm laser excitation and visualized with the appropriate long pass filters using a 20X/0.95 NA water objective. A 512 X 512 pixel back-thinned EMCCD camera (Model C9100-13; Hamamatsu Corp., Hamamatsu City, Japan) was used for fluorescence detection. 3–5 vessels (17–40 μm diameter) were recorded for 1 min and data averaged per mouse. Rolling leukocytes were classed as those that moved at a velocity less than that of an erythrocyte down a 100 μm segment of vessel. Adherent cells were classed as those that were stationary for 30 s or longer within the 100-μm segment of vessel [17]. Data are shown as mean ± SEM. For comparisons between two groups, an unpaired Student's *t*-test was performed (GraphPad Prism). A *P* value of ≤ 0.05 was considered significant. Exclusion criteria were established prior to initiation of the study. A total of 90 animals were successfully used in 32 cohorts. Four statistical outliers were identified using the Grubbs’ test and were removed.

### Cytokine measurements

To delineate the importance of leukocyte–cerebral endothelial cell interactions in initiating neuroimmune activation in the brain, we measured cortical cytokine levels 7 days after DSS treatment. On day 4 and 6 of DSS treatment, the control group (*n* = 6) was administered sterile phosphate-buffered saline (PBS) 10 mL/kg, IP while the DSS-treated mice were given either control IgG2a antibody (200 μg/mouse, IP; Bio X Cell; catalog #BE0089, *n* = 6), or anti-α4β7 integrin antibody (200 μg/mouse, IP; Bio X Cell; catalog #BE0034, *n* = 6) to investigate the role of integrins in cytokine changes in the brain. On day 5, DSS administration was stopped, and all mice were given tap water. On the morning of day 7, mice were anesthetized with isoflurane and transcardially perfused with cold PBS buffer for 5 min while under anesthesia. The cortex (consisting of motor, somatosensory, and parietal cortices) was microdissected using a published protocol [46] and immediately snap-frozen in liquid nitrogen and stored at − 80 °C until processing. The isolated cortical tissue was homogenized using a Micro-Tube homogenizer in tissue protein extraction buffer (RIPA buffer containing protease inhibitor cocktail). The homogenate was centrifuged at 15,000×*g* for 15 min at 4 °C, and supernatants were collected and stored at − 20 °C until analysis. Mouse cytokines (interferonγ, IL-1β, colony stimulating factor 2, IL-2, IL-4, IL-6, IL-10, IL-12(p70), CCL2, TNF) were simultaneously measured in tissue homogenate samples using a mouse MILLIPLEX kit (Millipore, Burlington, MA, USA) according to the manufacturer's protocol. The multiplexing analysis was performed using the Luminex 100 system (Luminex®, Austin, TX, USA) by Eve Technologies Corporation (Calgary, AB, Canada). Total protein concentration in tissue homogenates was quantified using a BCA Protein Assay kit (Catalog # 23225, ThermoFisher) according to the manufacturer's instructions. Results were expressed as pg of analyte/mg of protein. All data are shown as mean ± SEM of 6 mice per group. For comparisons between groups, an analysis of variance (ANOVA) followed by the Student–Newman–Keuls post hoc test was performed (GraphPad Prism). A *P* value of ≤ 0.05 was considered significant. Exclusion criteria were established prior to initiation of the study. The Grubbs’ test was used to identify and exclude potential statistical outliers.

### Intracerebroventricular (ICV) cannulation and infusion

To assess whether blocking elevated IL-1β levels in the brain would alter behavior we administered IL-1ra intracerebroventricularly. Mice were anesthetized with isoflurane and a guide cannula (23G, 8 mm) was implanted under stereotaxic guidance above the right lateral ventricle (from Bregma: + 0.5 mm, lateral: + 1.0 mm, depth: + 1.4 mm). Mice were given analgesic treatment (buprenorphine, provided by the Health Science Animal Resource Centre, University of Calgary, AB, Canada, 0.05 mg/kg subcutaneously before and after the surgery) and allowed to recover for 5 days, after which DSS was administered in drinking water for another 5 days as described above. Body weight was recorded pre- and post-surgery, as well as during and after DSS administration/ICV infusions.

At the onset of DSS administration, both control and DSS-treated mice were infused ICV (0.5 μL/30 s) with IL-1ra (2 μg/2 μL, Catalog #280-R, R&D Systems, Minneapolis, MN, USA) or vehicle (sterile PBS with 0.1% bovine serum albumin [BSA], 2 μL). This was repeated on day 2 and day 4 of DSS treatment, and the day after termination of DSS administration (day 6). Approximately 24 h after the last infusion, mice were placed in the elevated plus maze to test for anxiety-related behavior.

### Elevated plus maze (EPM)

The EPM was used for assessment of anxiety-related behavior, as we have done previously [19, 47]. Our EPM consisted of two open (6 × 30 cm, 70 lx) and two closed (6 × 30 × 15 cm, 20 lx) arms radiating from a central platform (6 × 6 cm, 55 lx) to form a plus-shaped figure. The maze was elevated 50 cm above the floor. Each mouse was placed on the central platform facing a closed arm and allowed to explore the maze for 5 min. The 5-min test period was recorded by means of a video camera and later analyzed using TopScanTM 2.0 software (Clever Sys Inc., Virginia, USA). This allowed for the calculation of the percentage time spent in the arms as well as the total distance travelled, which was deemed measures of anxiety and locomotion, respectively. The maze was thoroughly cleaned before each test.

### Data presentation and statistics for EPM

Each data set consists of experiments carried out in control (PBS, with 0.1% BSA) or IL-1ra-treated DSS and control mice from 2 cohorts of animals over a period of 3 months. Data are shown as individual data points as well as mean ± SEM. For statistical analysis, GraphPad Prism was used, and differences compared between control- vs DSS-treated animals by a 2-way ANOVA (factor treatment × drug), followed by Bonferroni post hoc test for % time in the open arms, closed arms and total distance travelled. Exclusion criteria were established prior to initiation of the study. A total of 37 animals were used in 2 cohorts. One mouse was eliminated due to technical difficulties and lack of video, while 3 outliers were identified using the Grubbs’ test. A *P* value of ≤ 0.05 was considered statistically significant.

## Results

We conducted the majority of our studies in female mice. We determined that mice had colitis based on the degree of macroscopic damage, using previously published methods [19, 23, 48]. In female mice, the macroscopic damage score after DSS administration was significantly increased (4.1 ± 0.3; *n* = 10; *t* = 10.6, *df* 18, *P* < 0.001) compared to their respective controls (0.4 ± 0.1, *n* = 10). Male mice treated identically have slightly higher macroscopic damage scores than (4.9 ± 0.4; *n* = 8; *t* = 6.9, *df* 10, *P* < 0.001), compared to their respective controls (0.5 ± 0.3, *n* = 4). Macroscopic damage scores for all groups used in these studies are shown in Additional file 1: Table S1.

**Fig. 1:**
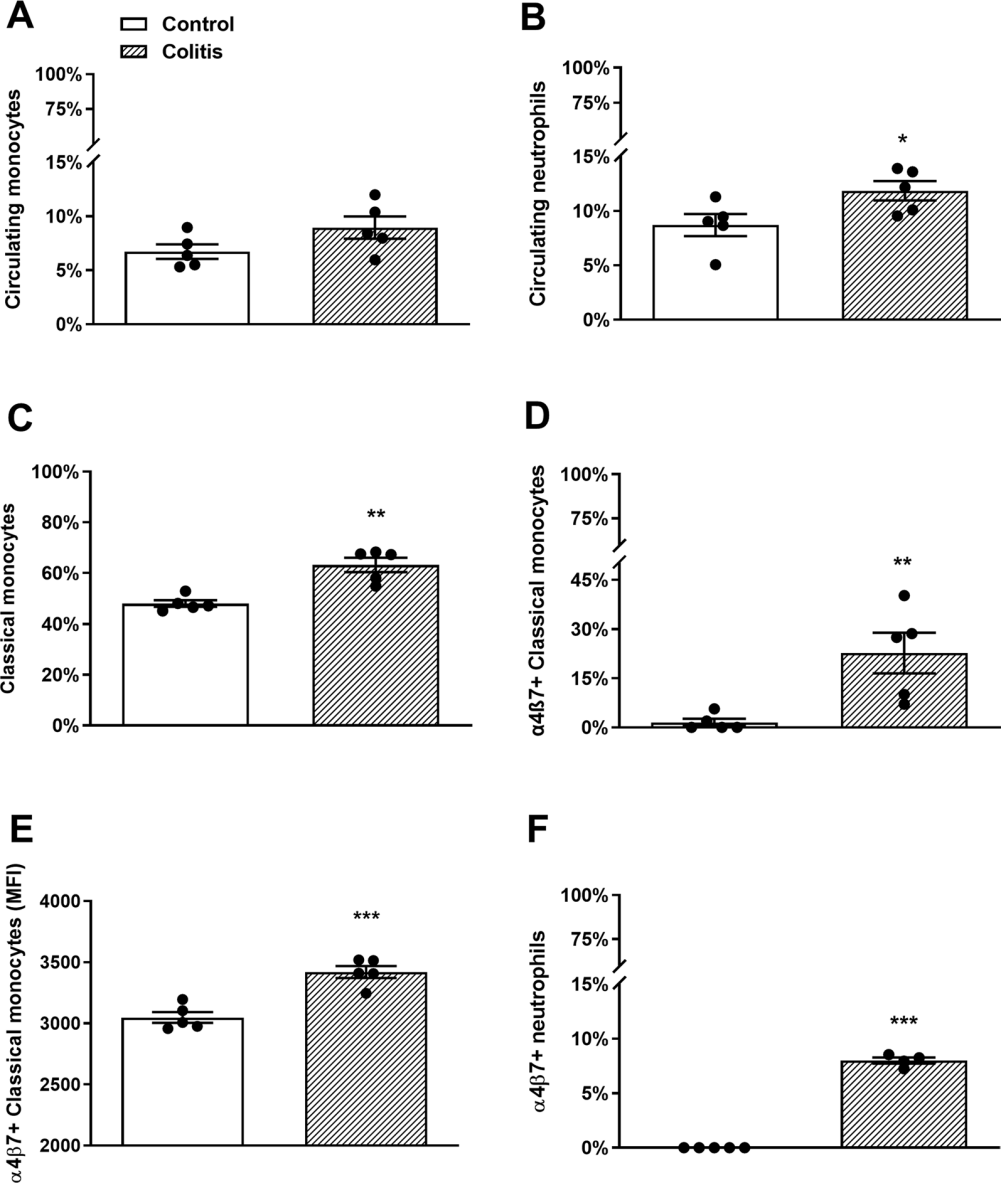
Colitis increases the percentage of classical monocytes and induces the upregulation of α4β7 integrin on circulating classical monocytes and neutrophils. The expression of α4β7 integrin on the surface of circulating peripheral blood monocytes and neutrophils from control and colitic female mice was assessed by multicolor flow cytometry. **A**, **B** Proportions of neutrophils and monocytes, represented as a percentage of total leukocytes, were similar in blood of DSS colitis mice and controls (monocytes, *t* = 1.8, *df* 8, *P* = 0.11; neutrophils, *t* = 0.2.4; *df* 8, *P* = 0.047, *n* = 5 mice/group). **C** Higher percentages of total classical monocytes were detected in the peripheral blood of colitic mice compared to control group (*t* = 4.9, *df* 8, ***P < 0.001; *n* = 5 mice/group). **D**, **E** There was a significant upregulation of α4β7 expression on the surface of classical monocytes in colitic mice. **D** Percentage of α4β7 + classical monocytes (*t* = 3.4, *df* 8, **P = 0.01; *n* = 5 mice/group). **E** Cell surface α4β7 expression on classical monocytes expressed as mean fluorescent intensity *t* = 5.6, *df* 8, ***P < 0.001; *n* = 5 mice/group). **F** Circulating blood neutrophils of control mice expressed very low levels of α4β7 which increased significantly in colitic mice (*t* = 32.7, *df* 8, ***P < 0.001; *n* = 5 mice/group)

It has previously been shown that neutrophils and monocytes traffic to the brain in DSS colitis [20]. To determine a possible mechanism, we first examined the expression of α4β7 integrin on circulating peripheral blood monocytes and neutrophils from control and DSS-treated female mice, 7 days after the induction of colitis. In accordance with previously published data [49], mice with DSS colitis showed significantly higher proportions of circulating neutrophils, compared with control animals. However, no change in the proportion of circulating monocytes was identified in colitic animals (Fig. 1, Additional file 1: Fig. S1).

Sequential gating was then used for the flow cytometric identification of α4β7 expressing monocytes and neutrophils from peripheral blood (Fig. 1). The percentage of classical monocytes was significantly increased in peripheral blood of mice with DSS colitis and the percentage of circulating inflammatory Ly6C^hi^ monocytes expressing α4β7 integrin was also significantly increased (Fig. 1). In contrast, a low level of α4β7 expression was found on neutrophils. The frequency of α4β7-positive neutrophils increased slightly in the peripheral blood of DSS colitis mice compared with controls (Fig. 1). Some signal bleed-over from the Ly6C channel may have occurred, therefore very low expression levels of α4β7 may have not been detected.

**Fig. 2:**
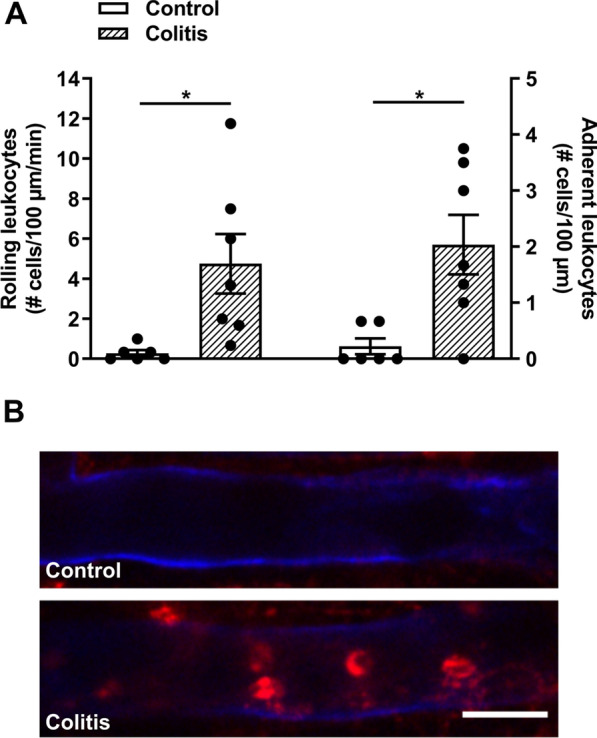
Colitis induces the rolling and adhering of leukocytes in cerebral endothelial cells. Intravital microscopy was performed using a spinning disc confocal microscope with a 20X/0.95 NA water objective. Videos were captured and analyzed to identify rolling and adhering leukocytes in control and colitic female mice. **A** Colitic mice showed a significant increase in rolling (*t* = 2.8, *df* 11, *P = 0.02; *n* = 6–7 mice/group) and adhering (*t* = 3.1, *df* 11, *P = 0.01; *n* = 6–7 mice/group) leukocytes on cerebral endothelial cells. **B** Representative images of intravital imaging. CD31 was used to label cerebral endothelial cells (blue), Rho6G was used to label leukocytes (red). Scale bar: 25 µm

We next used intravital microscopy to investigate the recruitment of immune cells to the cerebral vasculature of female mice with colitis [17, 20]. An increase in the number of rolling and adherent leukocytes on cerebral endothelial cells was observed (Fig. 2 and Additional file 2). In male mice with colitis, a similar increase in the number of rolling and adherent leukocytes along cerebral endothelial cells was measured (Additional file 1: Fig. S4).

**Fig. 3:**
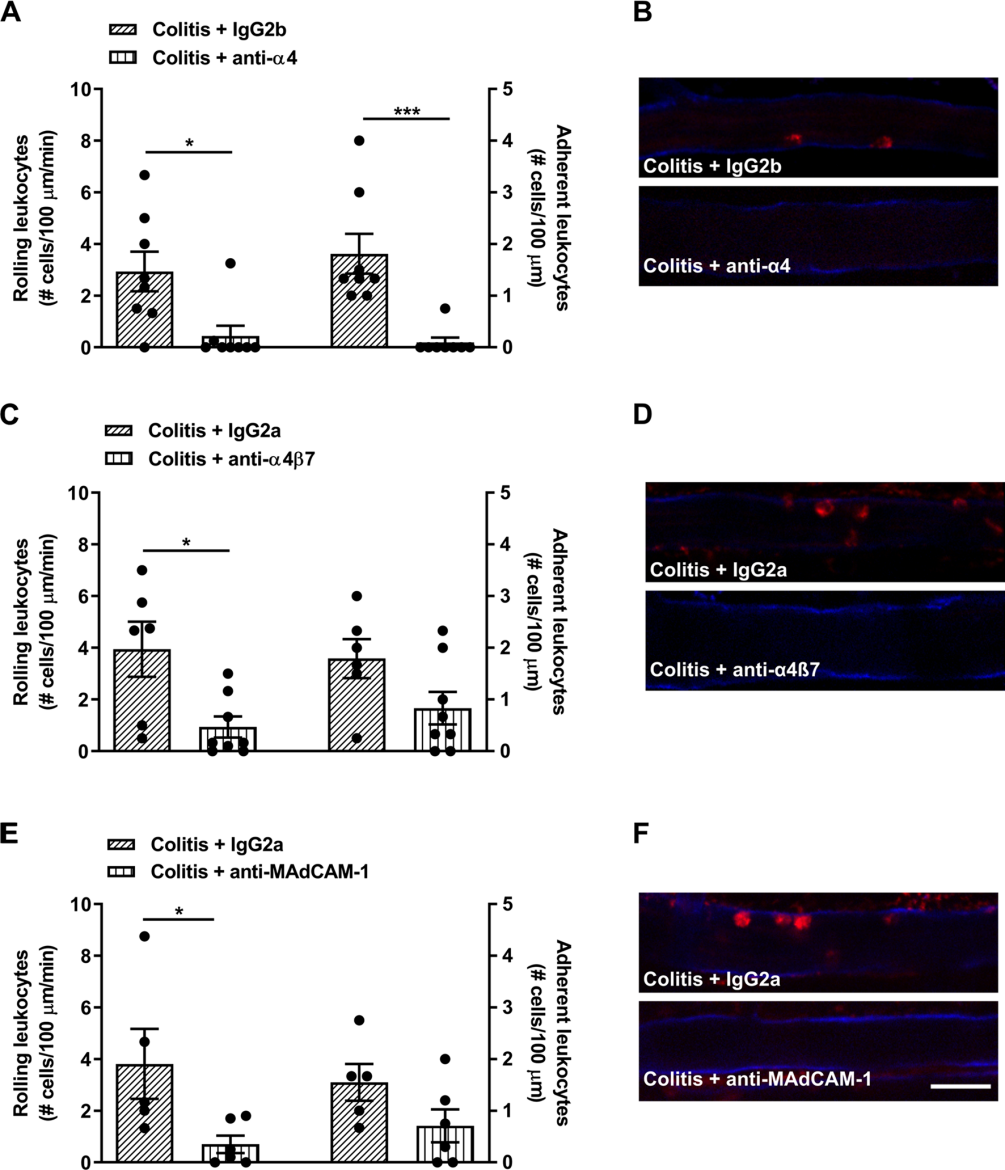
Anti-integrins block the rolling and adhering of leukocytes on cerebral endothelial cells during colitis. Intravital microscopy was used to identify rolling and adhering leukocytes in colitic female mice treated with an anti-integrin or an isotype control 18–22 h prior to imaging. **A** Anti-α4 integrin significantly reduced rolling (*t* = 2.9, *df* 14, *P = 0.01, *n* = 8 mice/group) and adhering (*t* = 4.3, *df* 14, ***P < 0.001, *n* = 8 mice/group) leukocytes in colitic mice compared to isotype-treated colitic controls. **B** Representative images from intravital microscopy. CD31 was used to label cerebral endothelial cells (blue), Rho6G was used to label leukocytes (red). **C** Anti-α4ß7 significantly reduced rolling leukocytes in colitic mice (*t* = 2.9, *df* 12, *P = 0.01, *n* = 6–8 mice/group) but not adhering (*t* = 2.0, *df* 12, *P* = 0.07, *n* = 6–8 mice/group) compared to isotype-treated colitic controls. **D** Representative images from intravital microscopy. CD31 was used to label cerebral endothelial cells (blue), Rho6G was used to label leukocytes (red). **E** Anti-MAdCAM-1 significantly reduced rolling leukocytes in colitic mice (*t* = 2.4, *df* 9, *P = 0.04, *n* = 5–6 mice/group) but not adhering (*t* = 1.8, *df* 9, *P* = 0.11, *n* = 5–6 mice/group) compared to isotype-treated colitic controls. **F** Representative images from intravital microscopy. CD31 was used to label cerebral endothelial cells (blue), Rho6G was used to label leukocytes (red). Scale bar for **B**, **D**, **F**: 25 µm

We then investigated the involvement of integrins in the mechanism of recruitment of leukocytes to cerebral endothelial cells in colitis. Treatment with anti-α4 integrin in mice with colitis 18–22 h prior to the imaging experiment significantly reduced the number of rolling and adherent leukocytes compared to colitic mice treated with the isotype control (Fig. 3A, B), without altering the degree of colitis (Additional file 1: Table S1). Similarly, colitic mice treated with anti-α4β7 displayed a significant reduction in the number of rolling leukocytes along cerebral endothelial cells (Fig. 3C, D) and a tendency for a reduction in adherent leukocytes (Fig. 3C; Colitis + IgG2a: 1.8 ± 0.8 cells/100 µm/min; colitis + anti-α4β7: 0.4 ± 0.3 cells/100 µm/min; *t* = 2.0, *df* 14, *P* = 0.07). The α4β7 integrin binds to the adhesion molecule MAdCAM-1 expressed on endothelial cells. In mice with colitis treated with a monoclonal antibody against MAdCAM-1, the number of rolling leukocytes along cerebral endothelial cells was also significantly reduced (Fig. 3E, F, Additional file 3), and a tendency for a reduction in adherent leukocytes was also observed (Fig. 3E; Colitis + IgG2a: 1.6 ± 0.4 cells/100 µm/min; colitis + anti-MAdCAM-1: 0.7 ± 0.3 cells/100 µm/min; *t* = 1.8, *df* 9, *P* = 0.11).

**Fig. 4:**
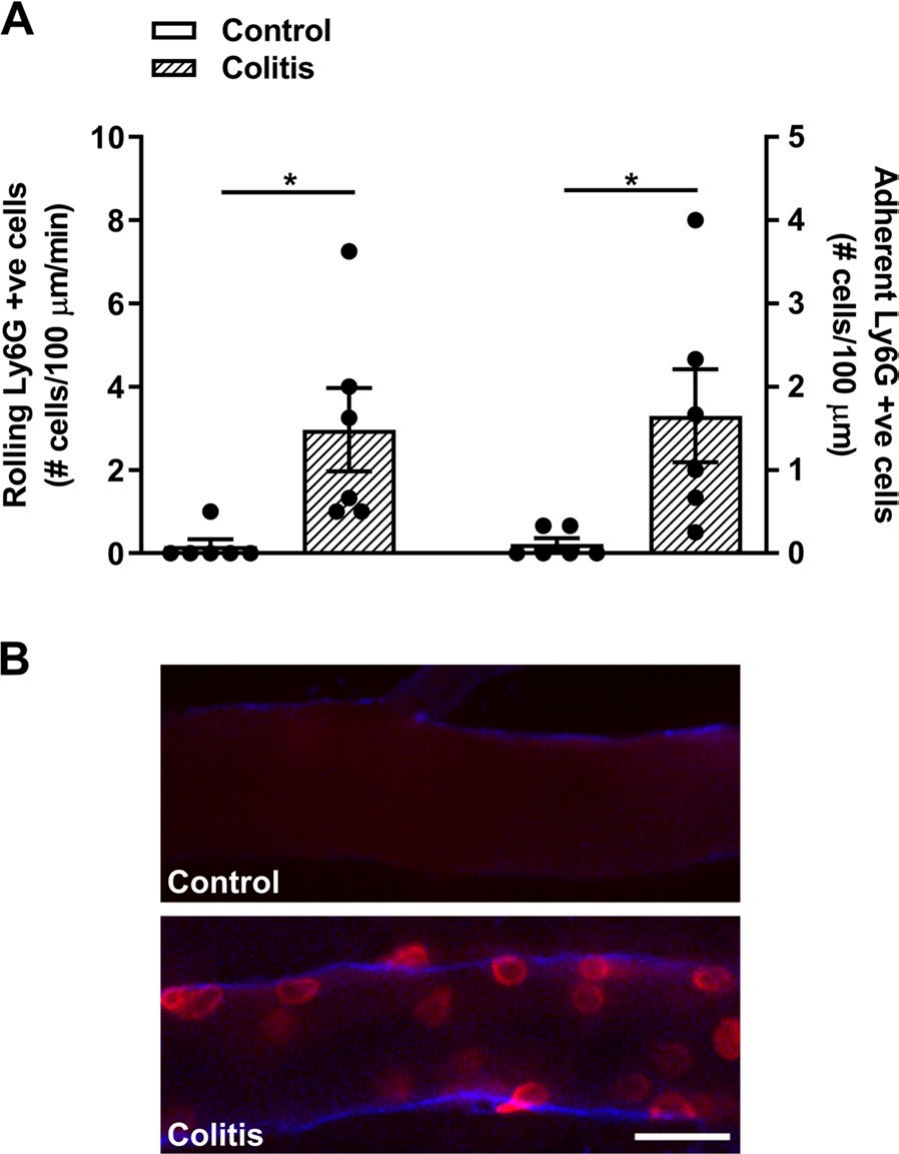
Colitis induces the rolling and adhering of neutrophils on cerebral endothelial cells during colitis. Intravital microscopy was used to identify rolling and adhering neutrophils (Ly-6G positive cells) in control and colitic female mice. **A** Colitis significantly increased the rolling (*t* = 2.8, *df* 10, *P = 0.01; *n* = 6 mice/group) and adhering (*t* = 2.7, *df* 10, *P < 0.02; *n* = 6 mice/group) neutrophils in colitic mice compared to controls. **B** Representative images from intravital microscopy. CD31 was used to label cerebral endothelial cells (blue), Ly6G was used to label neutrophils (red). Scale bar: 25 µm

**Fig. 5:**
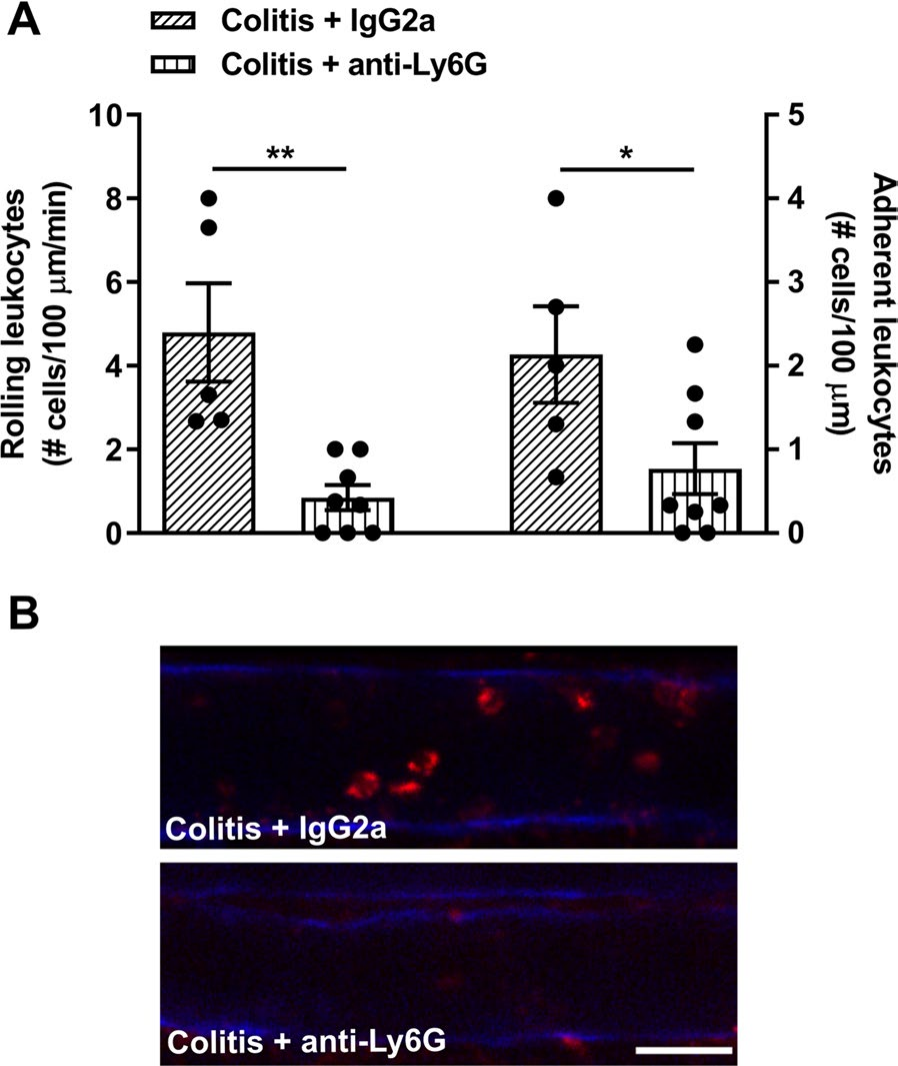
Blocking neutrophils reduces the rolling and adhering of leukocytes on cerebral endothelial cells during colitis. Intravital microscopy was used to visualize the rolling and adhering of leukocytes in colitic female mice treated with anti-Ly6G to block neutrophils compared to isotype controls. **A** Anti-Ly6G significantly reduced both rolling (*t* = 4.0, *df* 11, **P < 0.01; *n* = 5–8 mice/group) and adhering (*t* = 2.3, *df* 11, *P = 0.04; *n* = 5–8 mice/group) leukocytes in colitic mice compared to isotype-treated colitic controls. **B** Representative images from intravital microscopy. CD31 was used to label cerebral endothelial cells (blue), Rho6G was used to label leukocytes (red). Scale bar: 25 µm

In order to investigate the interaction of circulating neutrophils with cerebral endothelial cells in colitis we used a PE-conjugated Ly6G antibody to visualize neutrophils during intravital microscopy. In female mice with colitis, the number of Ly6G-PE positive cells interacting with cerebral endothelial was greater than in control mice (Fig. 4, Additional file 4). These observations were confirmed in male mice with colitis (Additional file 1: Fig. S4). To further examine their involvement, we used anti-Ly6G to deplete neutrophils in mice with colitis. Using flow cytometry, we confirmed that the neutrophils were depleted following an injection 200 µg of anti-Ly6G (Additional file 1: Fig. S2). Intravital microscopy showed that the number of both rolling and adherent leukocytes interacting with the cerebral endothelial cells of mice injected with anti-Ly6G with colitis was markedly reduced compared to colitic mice injected with isotype IgG control (Fig. 5).

**Fig. 6:**
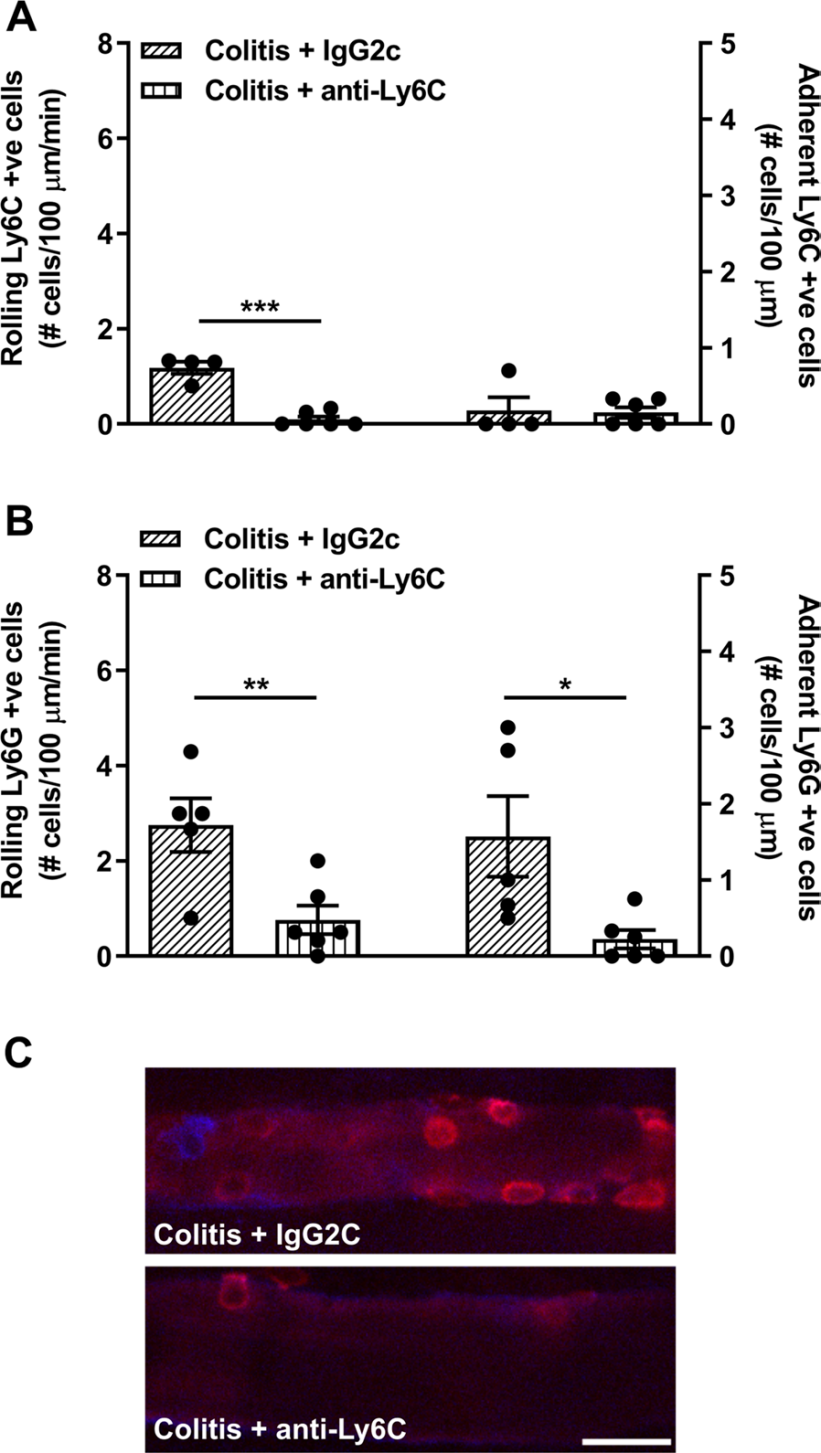
Anti-Ly6C depletion of monocytes reduces the rolling and adhering of neutrophils on cerebral endothelial cells during colitis. Intravital microscopy was used to visualize the rolling and adhering of neutrophils and monocytes in colitic female mice treated with anti-Ly6C to block monocytes or treated with an isotype control. **A** Colitic mice treated with anti-Ly6C significantly reduced rolling (*t* = 8.5, *df* 8, ***P < 0.001; *n* = 4–6 mice/group) but not adhering (*t* = 0.1, *df* 8, *P* = 0.89; *n* = 4–6 mice/group) monocytes (Ly6C positive cells) compared to colitic mice treated with an isotype control. **B** Anti-Ly6C treatment that reduces monocytes, significantly reduced rolling (*t* = 3.3, *df* 9, **P = 0.009; *n* = 5–6 mice/group) and adhering (*t* = 2.7, *df* 9, *P = 0.02, *n* = 5–6 mice/group) neutrophils in colitic mice compared to colitic mice treated with an isotype control. **C** Representative images from intravital microscopy. Ly6C was used to label monocytes (blue), Ly6G was used to label neutrophils (red). Scale bar: 25 µm

We then extended these findings to examine the role of monocytes, since neutrophils themselves have low levels of α4β7 expression (Fig. 1). In order to investigate the interaction of circulating monocytes with cerebral endothelial cells in colitis, we used an APC-conjugated Ly6C antibody to visualize monocytes during intravital microscopy (Fig. 6). We then used anti-Ly6C to deplete monocytes in mice with colitis. Using flow cytometry, we confirmed depletion of Ly6C^hi^ classical monocytes, but not neutrophils (Additional file 1: Fig S3). The number of rolling and adherent Ly6C-APC positive cells, (i.e., monocytes), was reduced in mice treated with anti-Ly6C (Fig. 6). We then treated mice with anti-Ly6C to deplete monocytes and examined the number of Ly6G-PE-positive cells (i.e., neutrophils), interacting with cerebral endothelial cells. Remarkably, we found the number of rolling and adhering neutrophils was significantly reduced when mice were treated with anti-Ly6C compared to those treated with the isotype control (Fig. 6, Additional file 5).

**Fig. 7:**
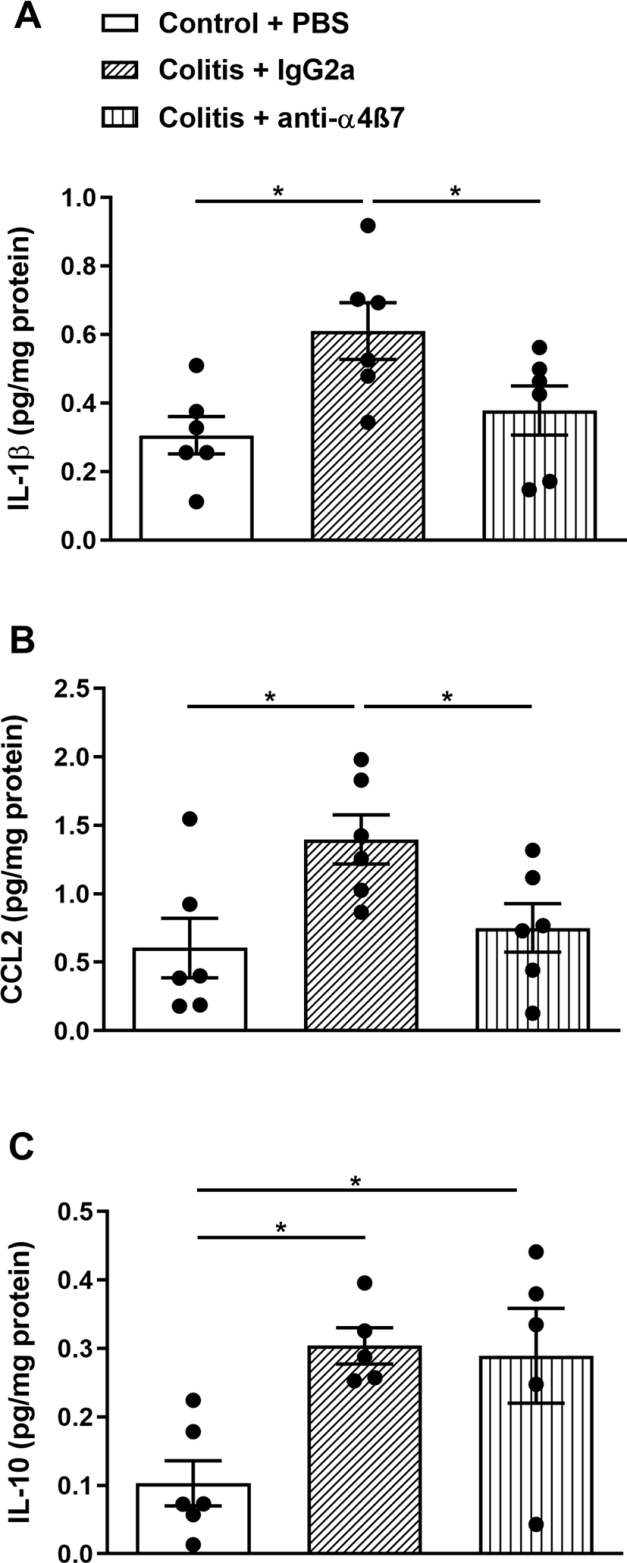
In vivo neutralization of α4β7 integrin modulates colitis-induced increases in cortical cytokine levels. To address the possible role of the α4β7 integrin in initiating neuroimmune activation, cortical cytokine levels of colitic female mice were assessed after anti-α4β7 treatment. Increased cortical cytokines levels of **A** IL-1β and **B** CCL2 induced by colitis were significantly reduced by anti-4β7 integrin antibody treatment (*F*(2, 15) = 4.79, *P = 0.02 and *F*(2, 15) = 5.08, *P = 0.02, respectively; colitis + anti-α4β7 vs. all other groups, one-way ANOVA; *n* = 6 mice per group). **C.** α4β7 integrin neutralization did not alter colitis-induced changes in cortical IL-10 levels (*F*(2, 13) = 6.45, *P = 0.01; colitis + anti-α4β7 and colitis + isotype vs. control group, one-way ANOVA; *n* = 6 mice per group)

In order to determine if leukocyte–cerebral endothelial cell interactions initiate neuroimmune activation, we measured cortical cytokine levels in DSS-treated mice. It has previously been shown that IL-1β levels are increased in the cortex of DSS-treated mice [20]. We confirmed and extended these findings by demonstrating that treatment with anti-α4β7 significantly reduced IL-1β levels (Fig. 7A). In addition, we measured C–C motif chemokine ligand 2 (CCL2), since this has previously been shown to be elevated in the brain in a mouse model of immune-mediated liver inflammation [45], and is a monocyte chemoattractant. CCL2 was significantly elevated in the brain in colitic mice, and treatment with anti-α4β7 significantly reduced CCL2 levels (Fig. 7B). In contrast, other cytokines that were elevated in the brain in mice with colitis (colony stimulating factor 2, IL-2, IL-6, IL-12(p70) [Additional file 1: Table S2] and IL-10, Fig. 7C) were unaffected by treatment with anti-α4β7.

**Fig. 8:**
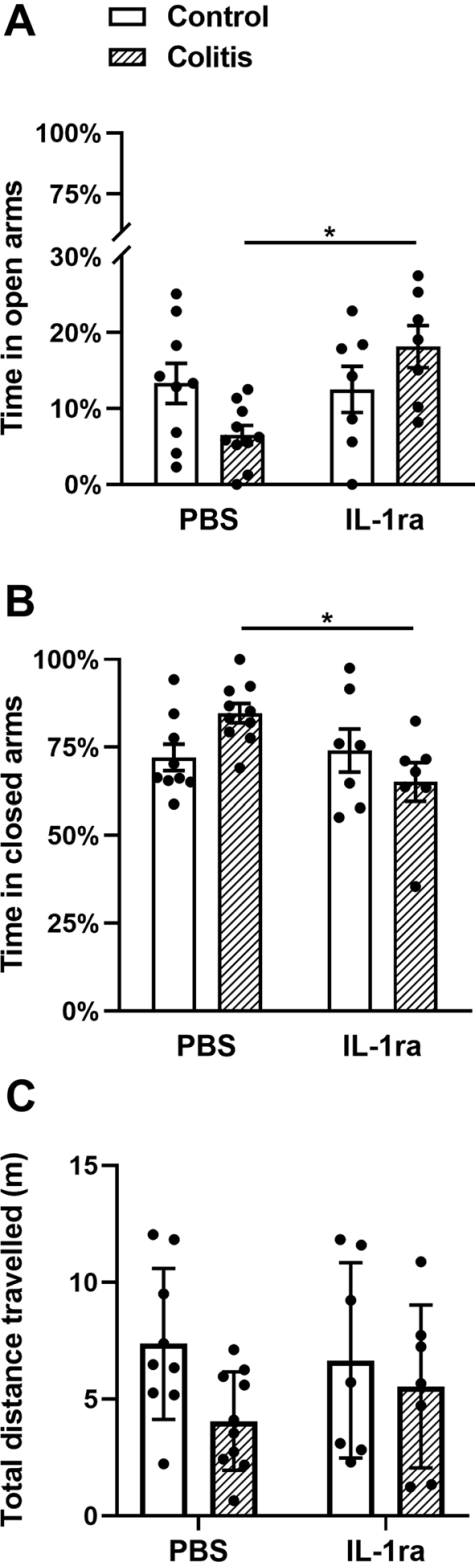
Interleukin-1 receptor antagonist (IL-1ra) reduces the anxiety-like phenotype in colitic mice but does not affect locomotion. To investigate the effect of colitis and IL-1ra on behavior, control and colitic female mice were implanted with intracerebroventricular cannulas above the right lateral ventricle and infused with either PBS or IL-1ra. At peak colitis, mice were assessed for anxiety-like behavior using the elevated plus maze. **A** In colitic mice, IL-1ra significantly increased the percentage time spent in the open arms of the maze (*F*(1, 29) = 5.0, *P = 0.01; *n* = 7–10 mice/group, two-way ANOVA). **B** In colitic mice, IL-1ra significantly decreased the percentage time spent in the closed arms of the maze (*F*(1, 29) = 4.0, *P = 0.021; *n* = 7–10 mice/group, two-way ANOVA) compared to colitic mice infused with PBS, indicating a decrease in anxiety-like behavior. **C** Total distance traveled in the elevated plus maze remained unchanged across each treatment condition (*F*(1, 29) = 0.12, *P* = 0.73; *n* = 7–10 mice/group)

Finally, to determine the functional significance of the elevated levels of IL-1β, we assessed anxiety-like behavior using the elevated plus maze. We were unable to block the reduced anxiety-like behavior by treating animals with either anti-α4β7 or anti-MAdCAM-1 antibodies after initiating colitis (Additional file 1: Fig. S5). We reasoned that elevations in central cytokines earlier in the course of disease may have already initiated the changes in the CNS that lead to altered behavior. Because we cannot treat mice earlier with peripherally administered anti-α4β7 or anti-MAdCAM-1 antibodies as they will alter the degree of colitis [50–53], we therefore used a strategy of blocking IL-1β with an ICV infusion of the IL-1 receptor antagonist (IL-1ra) from day 2. Administration of IL-1ra significantly increased the amount of time spent in the open arms (*F*(1,29) = 5.0, *P* = 0.03), and subsequently decreased closed arm time (*F*(1,29) = 4.0, *P* = 0.05) in animals with colitis, compared with their respective PBS-treated controls (Fig. 8). Furthermore, a significant interaction effect was evident when we examined open arm (factor treatment × drug: *F*(1,29) = 6.6, *P* = 0.02) and closed arm time (factor treatment × drug: *F*(1,29) = 6.0, *P* = 0.02). Post hoc comparisons revealed that colitic mice treated with IL-1ra spent more time in the open arms (Fig. 8A), and less time in the closed arms (Fig. 8B), when compared with PBS-treated DSS mice. IL-ra treatment did not affect overall distance travelled in the maze (Fig. 8C).

## Discussion

Patients with IBD exhibit a higher prevalence of cognitive, behavioral and emotional disorders when compared to the general population [6–10]. However, the mechanisms underlying these changes in the brain remain poorly understood. Here using an acute model of colitis, we identified a novel mechanism whereby classical monocytes within the circulation adhere to the cerebral endothelium, through integrin α4β7-MAdCAM-1 interactions, which directs the recruitment of neutrophils to the brain vasculature, leading to increased expression of IL-1β that mediates anxiety-like behavior. These exciting observations may explain, at least in part, the behavioral benefits of anti-integrin monoclonal antibody, vedolizumab in patients with IBD [38] and identify new potential targets for the treatment of the maladaptive behaviors that commonly affect IBD patients, even when their disease is in clinical remission.

In contrast to physical symptoms of IBD, the psychological manifestations of these disorders demonstrate only modest improvements over long-term follow-up [54, 55], contribute to poor outcomes [56], and are associated with increased mortality [57]. Current therapies that alleviate physical symptoms and induce disease remission have little impact on IBD-associated psychological symptoms [58–61]. In fact, these changes often go undiagnosed and are poorly managed [62–64]. Despite their high prevalence, the etiology of such symptoms in patients is poorly understood. However, to mediate changes within the CNS that alter behavior, communication pathways must exist between the inflamed gut and the brain. To date, neural, humoral (i.e., circulating gut-derived mediators) and microbial signaling pathways from the gut to the brain have been most widely studied as underlying mechanisms regulating colitis-associated behavioral changes [65–70]. Our group has previously identified a role for the cerebral recruitment of activated classical monocytes from the circulation to the brain as an important mechanism linking liver inflammation to neuroinflammation, and subsequent changes in brain function and behavior [16, 17, 45]. Specifically, we showed that the rolling and adhesion of classical monocytes to cerebral endothelium enhanced levels of proinflammatory cytokines and the chemokine CCL2 in the brain, which critically regulated the subsequent migration of monocytes into the brain to drive inflammation-associated development of maladaptive behaviors [16, 17, 45]. In our current study, we have extended our previous observations in experimental liver disease to a model of acute colitis and found important differences between these models. Previously, we showed that monocytes were exclusively recruited to the brain vasculature in mice with liver inflammation, not neutrophils, and this recruitment led to increased levels of tumor necrosis factor (TNF) and CCL2 within the CNS [45]. Our current findings suggest that acute gut and liver inflammatory processes differentially impact neuroimmune responses within the brain. Specifically, neutrophils, not monocytes, represent the predominant leukocyte recruited to the brain during acute colitis, and the resulting adhesive interactions with the cerebral endothelium drives an increased expression of CCL2 and IL-1β in the brain.

Our findings are in agreement with a previous intravital microscopy study in mice where it was demonstrated that leukocytes are recruited to the vasculature of the prefrontal cortex during the recovery phase of DSS colitis (7 days after discontinuation of DSS) [20]. In that study, rolling leukocytes consisted of similar numbers of neutrophils and monocytes, and classical monocytes were the predominant adherent leukocyte. We studied a more acute phase of colitis and found that at this time point neutrophils were the predominant leukocyte subtype, with monocytes representing only a minor cell population. These observations imply that recruitment of different leukocyte populations to the brain vasculature during peripheral inflammatory processes involving different organ systems or timelines, may differentially impact neuroimmune responses within the brain microvasculature.

The role of neutrophils recruited to the brain in colitis was the subject of a recent study in mice in the recovery phase of acute colitis (9 days after discontinuing DSS) [68]. Here it was shown that neutrophils, but not monocytes, regulate neuronal excitability and that the reduction in seizure thresholds was mediated by TNF. However, both neutrophils and monocytes were significantly elevated, both in this late acute phase and during chronic colitis [68]. Interestingly, infiltrating neutrophils were the source of TNF.

Classically, neutrophils are considered the first cells recruited to inflammatory sites and they subsequently mediate the recruitment of monocytes [71]. However, a number of studies have clearly shown that early monocyte recruitment is essential in some scenarios to promote subsequent neutrophil adhesion to endothelium and ultimate robust neutrophil recruitment into inflammatory sites [72–74]. This neutrophil adhesion-enhancing effect of monocytes at the level of the endothelium was shown to be mediated by early monocyte adhesion that induced endothelial cell activation and subsequently increased endothelial expression of neutrophil binding ligands ICAM-1 and E-selectin [75]. Moreover, this pro-neutrophil adhesive effect occurred at physiological circulating monocyte levels and did not depend on cell–cell interactions [75]. Similarly, we now show that monocyte recruitment critically regulates neutrophil recruitment to the brain in the setting of DSS colitis. However, in contrast to earlier observations, we found that monocyte–cerebral endothelial adhesive interactions regulate subsequent neutrophil recruitment via endothelial cell upregulation of MAdCAM-1 expression. Moreover, this cell adhesive process and neutrophil recruitment is critically dependent on α4β7-MAdCAM-1 interactions. The mechanism linking monocyte recruitment to subsequent neutrophil recruitment is currently unclear, but may lie in the production of a neutrophil chemoattractant that is enhanced during monocyte–endothelial adhesive interactions.

Previous work has shown that cerebral endothelial expression of adhesion molecules involved in leukocyte recruitment is upregulated in models of colitis in both rats and mice [76]. However, MAdCAM-1 is not thought to be expressed on murine cerebral endothelial cells either constitutively or in IL-10 knockout mice during inflammation [77, 78]. Nonetheless its expression can be induced in brain endothelial cells [79] and it has been demonstrated by electron microscopy in the inflamed spinal cord of mice [80]. In human brain, MAdCAM-1 has been cloned and various alternatively spliced variants have also been identified [81], but its function there remains to be determined. Further studies are required to determine where α4β7 expressing monocytes and MAdCAM-1 are interacting to prime the neutrophils that subsequently enter the brain in this model of colitis.

Inflammatory effector lymphocytes expressing α4β7 are recruited to the bowel in IBD patients, and this observation precipitated the development of α4β7 blockade using vedolizumab as an effective treatment strategy in IBD [34, 35]. However, it has become increasingly clear that α4β7 is also expressed on monocytes, in both mice and humans, and regulates the trafficking of both inflammatory and repair monocytes to the bowel [36, 37]. In keeping with these previous observations, we have identified a significant α4β7-expressing monocyte population in the peripheral blood of mice with DSS colitis. We did not find an impact of α4β7 blockade on DSS colitis severity at the time it was administered since colitis was already established.

In mice with liver inflammation, TNF played the central role in regulating the expression of maladaptive behaviors [17] and we have also demonstrated a role for TNF in regulating neuronal excitability in rats with colitis induced by trinitrobenzene sulphonic acid [27, 28]. As noted above, in the recovery phase of acute DSS colitis, TNF mediates changes neuronal hyperexcitability [68]. However, we found that IL-1β is elevated in the acute DSS model of colitis in mice, as it is in the hippocampus of mice with chronic DSS colitis [82]. The magnitude of the changes in IL-1β levels we observed are similar to that observed in the hippocampus in chronic colitis [82] and in febrile seizures [83]. Increased levels of the proinflammatory cytokine IL-1β in the brain induces a number of behavioral changes, including anxiety-like behaviors [84, 85], and inhibition of IL-1β-mediated effects in the brain attenuates these behavioral changes [86, 87]. The source of increased brain IL-1β levels in DSS-treated mice remains unknown. Potential sources include leukocytes that may have infiltrated into the brain [88, 89], microglia, astrocytes and neurons [90–92], with IL-1β produced from such cells interacting with IL-1R1 on neurons involved in the anxiety circuits and behaviors observed in the current study.

In accord with previous observations from our laboratories [19] and elsewhere [20–25], we show that mice with DSS colitis demonstrate anxiety-like behaviors measured using the elevated plus maze. As this test does not discriminate between anxiety-like behavior versus changes in impulsivity and engagement of risk behaviors [93], we recognize this as a limitation of the current study. In addition, similar to observations by Gadotti et al*.* [20], we show that DSS colitis also induces IL-1β expression in the brain. Here we provide novel information that this colitis-associated increase in IL-1β expression is driven by α4β7-mediated leukocyte adhesive interactions with cerebral endothelium. We were not able to block the reduced anxiety-like behavior by treating animals with anti-α4β7 or anti-MAdCAM-1 antibodies after initiating colitis, suggesting that elevations in central cytokines earlier in the course of disease may have already initiated the changes in the CNS. Nevertheless, by attenuating the development of anxiety-like behaviors in colitic mice by blocking IL-1β centrally we link our observations, albeit indirectly. Further studies are required to directly address the link between activation of the cerebral endothelium, elevated brain cytokines and changes in behavior. Based on previous work [26, 94–96], it is likely these involve glia (astrocytes and/or microglia) and alterations in hippocampal neurogenesis. Nevertheless, given the frequent association of maladaptive behavioral changes, including anxiety, in IBD patients [6, 7, 9], our new observations suggest that this leukocyte–cerebral endothelium–IL-1β axis may represent an opportunity to develop novel targets for treating IBD-associated behavioral alterations.

## Conclusions

In conclusion, by showing that α4β7 integrin-expressing monocytes direct the recruitment of neutrophils to the brain in acute colitis, we have identified a new mechanism that helps explain how peripheral inflammation alters activity in the CNS, which ultimately leads to changes in behavior. In this study, we examined anxiety-like behavior and demonstrated that this was mediated by the proinflammatory cytokine IL-1β. In future studies, other behaviors, such as depression and fatigue should be assessed to build a more comprehensive understanding of the role of this novel mechanism in mediating the behavioral comorbidities of colitis.

## Supplementary Information

**Additional file 1: Fig. S1.** Gating strategies for flow cytometric identification of α4β7 expressing monocytes and neutrophils in mouse blood. Gating proceeded as follows: exclusion of doublet cells followed by gating on forward scatter (FSC) and side scatter (SSC) areas to identify regions appropriate to define all live cells. Live cells were first gated on a CD3^+^ and CD3^−^ gate. Within the CD3^−^ gate, the population cells expressing the myeloid lineage marker CD11b were identified (density plot panel **A**). Within the CD11b^+^ subpopulation, neutrophils were identified as CD3^−^CD11b^+^ Ly6C^low^ Ly6G^+^ (density plot panel **B**). Monocytes were identified as CD3^−^CD11b^+^Ly6G^−^Ly6C^+^ and subdivided into two distinct subsets of classical monocytes (Ly6C^hi^) and non-classical (Ly6C^−^) monocytes (density plot panels **B** and **C**). Subsequently, α4β7 integrins positivity for each cell subpopulation was identified using an antibody that recognizes α4β7 heterodimeric complex based on the shift above the fluorescence-minus-one (FMO) controls (density plot panel **D**). Representative flow cytometry plots illustrating FMO controls for the gating strategy for α4β7 expression on circulating monocytes. Left panel shows the FMO control α4β7 expression results, and the right panel shows staining with full antibody panel. FMO boundaries separate true positive signals from negative signals by accounting for the spread of the negative population, as determined using the FMO control. Autofluorescence levels are affected by cell types and physiological conditions, which in turn can affect FMO controls. To mitigate the impact of any possible changes in autofluorescence levels as a result of changing the experimental conditions, the cells used in the control tubes, including the FMO controls, always included a mixture of cells that included all treatment groups. **Fig. S2.** The anti-Ly6G ab efficiently depleted neutrophils in C57BL/6J mice. Efficiency of the monoclonal antibody (mAb) anti-Ly6G (clone 1A8) to specifically deplete neutrophils in C57BL/6J mice was assessed using flow cytometry. The anti-Ly6G mAb (200 µg per mouse) efficiently depleted circulating neutrophils in vivo. Representative flow cytometry forward vs side scatter plots show the percentage of neutrophils in the total leukocyte population; isotype control treated (left panel) and anti-Ly6G-treated (right panel). The neutrophil gate is shown in the upper right box for each panel. **Fig. S3.** The anti-Ly6C antibody efficiently depleted classical monocytes but not neutrophils in C57BL/6J mice. The efficiency of the monoclonal antibody anti-Ly6C (100 µg per mouse) to specifically deplete classical monocytes in C57BL/6J mice was assessed using flow cytometry. Administration of anti-Ly6C efficiently depleted circulating classical monocytes but did not affect circulating neutrophils. **A** Representative flow cytometric histograms showing CD11b + Ly6G-Ly6Chi classical monocytes as a percentage of CD11b + cells; isotype control-treated (left panel), and anti-Ly6C-treated (right panel). **B** Representative flow cytometric histograms showing the percentage of Ly6G + neutrophils on CD11b + cells; isotype control treated (left panel) and anti-Ly6C-treated (right panel). **Table S1.** Macroscopic damage scores. **Fig. S4.** Colitis induces the rolling and adherence of leukocytes and the rolling of neutrophils along cerebral endothelial cells of male mice. Intravital microscopy was performed using a spinning disc confocal microscope. Videos were captured and analyzed to identify rolling and adhering of leukocytes in control and colitic mice. **A** Colitic male mice showed a significant increase in the rolling (*t* = 2.3, *df* 10, *p = 0.047, *n* = 5–8 mice/group) and adhering (*t* = 4.6, *df* 10, ***p < 0.001, *n* = 4–8 mice/group) of leukocytes in CECs. **B** Colitis significantly increases the rolling (*t* = 2.5, *df* 6, *p = 0.044; *n* = 4 mice/group) but not adhering (*t* = 1.2, *df* 6, p < 0.28; *n* = 4 mice/group) of neutrophils (Ly6G positive cells) in colitic male mice compared to controls. **Table S2.** Supplementary cytokine data table. **Fig. S5.** In vivo neutralization of α4β7 integrin or anti-MAdCAM-1 does not reduce anxiety-like phenotype in colitic mice. To investigate the effect of blocking α4β7 integrin or MAdCAM-1 on behavior, colitic female mice were assessed after anti-α4β7 or anti-MAdCAM-1 treatment mice. On day 4 and 6 of DSS treatment, the control group (*n* = 5) was administered sterile phosphate-buffered saline (PBS) 10 mL/kg, IP, while the DSS-treated mice were given either control IgG2a antibody (200 μg/mouse, IP; Bio X Cell; catalog #BE0089, *n* = 5), or anti-α4β7 integrin antibody (200 μg/mouse, IP; Bio X Cell; catalog #BE0034, *n* = 5) or on days 3 and 5 of DSS treatment, other mice were (*n* = 9) were administered sterile phosphate-buffered saline (PBS) 10 mL/kg, IP, while the DSS-treated mice were given either control IgG2a antibody (200 μg/mouse, IP; Bio X Cell; catalog #BE0089, *n* = 9), or anti-MAdCAM-1 (MECA-367; 200 μg/mouse; Bio X Cell; catalog #BE0035, *n* = 10) to investigate the role of integrins in behavioral changes. At peak colitis, mice were assessed for anxiety-like behavior using the elevated plus maze. **A** In colitic mice, anti-α4β7 did not significantly alter the percentage time spent in the open arms of the maze (F2, 12) = 0.18, *P* = 0.84; one-way ANOVA). **B** Similarly, anti-α4β7 did not significantly alter the percentage time spent in the closed arms of the maze (*F*(2, 12) = 3.0, *P* = 0.09; one-way ANOVA). **C** In colitic mice, anti-MAdCAM-1 did not significantly alter the percentage time spent in the open arms of the maze (F2, 25) = 0.64, *P* = 0.53; one-way ANOVA). **D** Similarly, anti-MAdCAM-1 did not significantly alter the percentage time spent in the closed arms of the maze (*F*(2, 25) = 0.69, *P* = 0.51; one-way ANOVA).

**Additional file 2.** Colitis induces the rolling and adhering of leukocytes along cerebral endothelial cells. Representative intravital microscopy videos of leukocytes (Rho6G positive cells) on cerebral endothelial cells in control and colitic animals. Colitis markedly increases the rolling and adhering of leukocytes. Frame rate = 7 frames per second, scale bar = 25 µm.

**Additional file 3.** Anti-α4β7 blocks the rolling and adhering of leukocytes along cerebral endothelial cells during colitis. Representative intravital microscopy videos of leukocytes (Rho6G positive cells) on cerebral endothelial cells in colitic animals treated with either anti-α4β7 or isotype control. Anti-α4β7 treatment diminishes the rolling and adhering of leukocytes during colitis. Frame rate = 7 frames per second, scale bar = 25 µm.

**Additional file 4.** Colitis induces the rolling and adhering of neutrophils along cerebral endothelial cells. Representative intravital microscopy videos of neutrophils (Ly6G + positive cells) on cerebral endothelial cells in control and colitic animals. Colitis markedly increases the rolling and adhering of neutrophils. Frame rate = 7 frames per second, scale bar = 25 µm.

**Additional file 5.** Depleting monocytes reduces the rolling and adhering of neutrophils along cerebral endothelial cells during colitis. Representative intravital microscopy videos of neutrophils (red; Ly6G + positive cells) and monocytes (blue; Ly6C + positive cells) on cerebral endothelial cells in colitic animals treated with either anti-Ly6C to deplete monocytes or isotype control. Anti-Ly6C treatment decreases the rolling and adhesion of neutrophils in colitic animals. Frame rate = 7 frames per second, scale bar = 25 µm.

## Data Availability

All data generated or analyzed during this study are included in this published article and the additional data files. The datasets used and/or analyzed during the current study are available from the corresponding author on reasonable request.

## Abbreviations

APC: Allophycocyanin
ANOVA: Analysis of variance
BSA: Bovine serum albumin
CCL2: C–C motif chemokine ligand 2
CNS: Central nervous system
DSS: Dextran sodium sulfate
GI: Gastrointestinal
IBD: Inflammatory bowel disease
ICV: Intracerebroventricular
IL: Interleukin
IL-1ra: Interleukin 1 receptor antagonist
IP: Intraperitoneal
MAdCAM-1: Mucosal addressin cell adhesion molecule-1
PBS: Phosphate buffered saline
PE: Phycoerythrin
SEM: Standard error of the mean
TNF: Tumor necrosis factor
