# Supplementary material for: Recruitment of α4β7 monocytes and neutrophils to the brain in experimental colitis is associated with elevated cytokines and anxiety-like behavior
Source: J Neuroinflammation. 2022 Apr 4;19:73. doi: 10.1186/s12974-022-02431-z (PMC8981853; doi:10.1186/s12974-022-02431-z)
Supplement: Supplementary file 1 — Additional file 1: Fig. S1. Gating strategies for flow cytometric identification of α4β7 expressing monocytes and neutrophils in mouse blood. Gating proceeded as follows: exclusion of doublet cells followed by gating on forward scatter (FSC) and side scatter (SSC) areas to identify regions appropriate to define all live cells. Live cells were first gated on a CD3+ and CD3− gate. Within the CD3− gate, the population cells expressing the myeloid lineage marker CD11b were identified (density plot panel A). Within the CD11b+ subpopulation, neutrophils were identified as CD3−CD11b+ Ly6Clow Ly6G+ (density plot panel B). Monocytes were identified as CD3−CD11b+Ly6G−Ly6C+ and subdivided into two distinct subsets of classical monocytes (Ly6Chi) and non-classical (Ly6C−) monocytes (density plot panels B and C). Subsequently, α4β7 integrins positivity for each cell subpopulation was identified using an antibody that recognizes α4β7 heterodimeric complex based on the shift above the fluorescence-minus-one (FMO) controls (density plot panel D). Representative flow cytometry plots illustrating FMO controls for the gating strategy for α4β7 expression on circulating monocytes. Left panel shows the FMO control α4β7 expression results, and the right panel shows staining with full antibody panel. FMO boundaries separate true positive signals from negative signals by accounting for the spread of the negative population, as determined using the FMO control. Autofluorescence levels are affected by cell types and physiological conditions, which in turn can affect FMO controls. To mitigate the impact of any possible changes in autofluorescence levels as a result of changing the experimental conditions, the cells used in the control tubes, including the FMO controls, always included a mixture of cells that included all treatment groups. Fig. S2. The anti-Ly6G ab efficiently depleted neutrophils in C57BL/6J mice. Efficiency of the monoclonal antibody (mAb) anti-Ly6G (clone 1A8) to specificall [file 12974_2022_2431_MOESM1_ESM.docx]

**ADDITIONAL FILE 1 (Figs and Tables)**

**
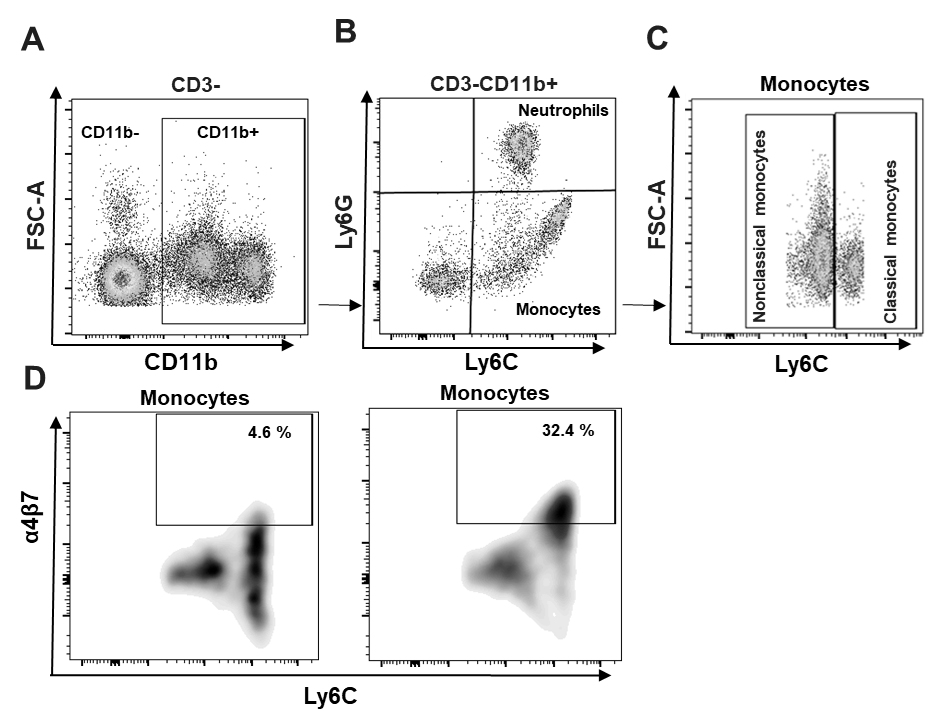
**

**Figure S1: Gating strategies for flow cytometric identification of α4β7 expressing monocytes and neutrophils in mouse blood.**

Gating proceeded as follows: exclusion of doublet cells followed by gating on forward scatter (FSC) and side scatter (SSC) areas to identify regions appropriate to define all live cells. Live cells were first gated on a CD3^+^ and CD3^-^ gate. Within the CD3^-^ gate, the population cells expressing the myeloid lineage marker CD11b were identified (density plot panel A). Within the CD11b^+^ subpopulation, neutrophils were identified as CD3^-^CD11b^+^ Ly6C^low^ Ly6G^+^ (density plot panel B). Monocytes were identified as CD3^-^CD11b^+^Ly6G^-^Ly6C^+^ and subdivided into two distinct subsets of classical monocytes (Ly6C^hi^) and non-classical (Ly6C^−^) monocytes (density plot panels B and C). Subsequently, α4β7 integrins positivity for each cell subpopulation was identified using an antibody that recognizes α4β7 heterodimeric complex based on the shift above the fluorescence-minus-one (FMO) controls (density plot panel D). Representative flow cytometry plots illustrating FMO controls for the gating strategy for α4β7 expression on circulating monocytes. Left panel shows the FMO control α4β7 expression results, and the right panel shows staining with full antibody panel. FMO boundaries separate true positive signals from negative signals by accounting for the spread of the negative population, as determined using the FMO control. Autofluorescence levels are affected by cell types and physiological conditions, which in turn can affect FMO controls. To mitigate the impact of any possible changes in autofluorescence levels as a result of changing the experimental conditions, the cells used in the control tubes, including the FMO controls, always included a mixture of cells that included all treatment groups.


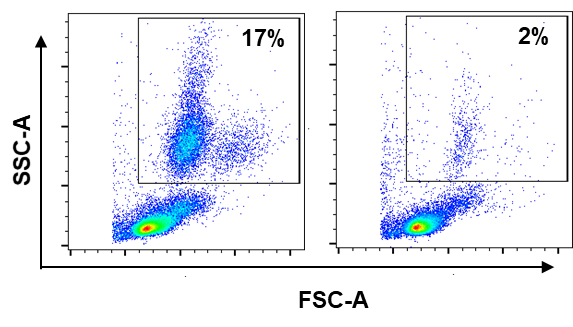


**Figure S2: The anti-Ly6G ab efficiently depleted neutrophils in C57BL/6J mice.**

Efficiency of the monoclonal antibody (mAb) anti-Ly6G (clone 1A8) to specifically deplete neutrophils in C57BL/6J mice was assessed using flow cytometry. The anti-Ly6G mAb (200 µg per mouse) efficiently depleted circulating neutrophils *in vivo*. Representative flow cytometry forward vs side scatter plots show the percentage of neutrophils in the total leukocyte population; isotype control treated (left panel) and anti-Ly6G-treated (right panel). The neutrophil gate is shown in the upper right box for each panel.


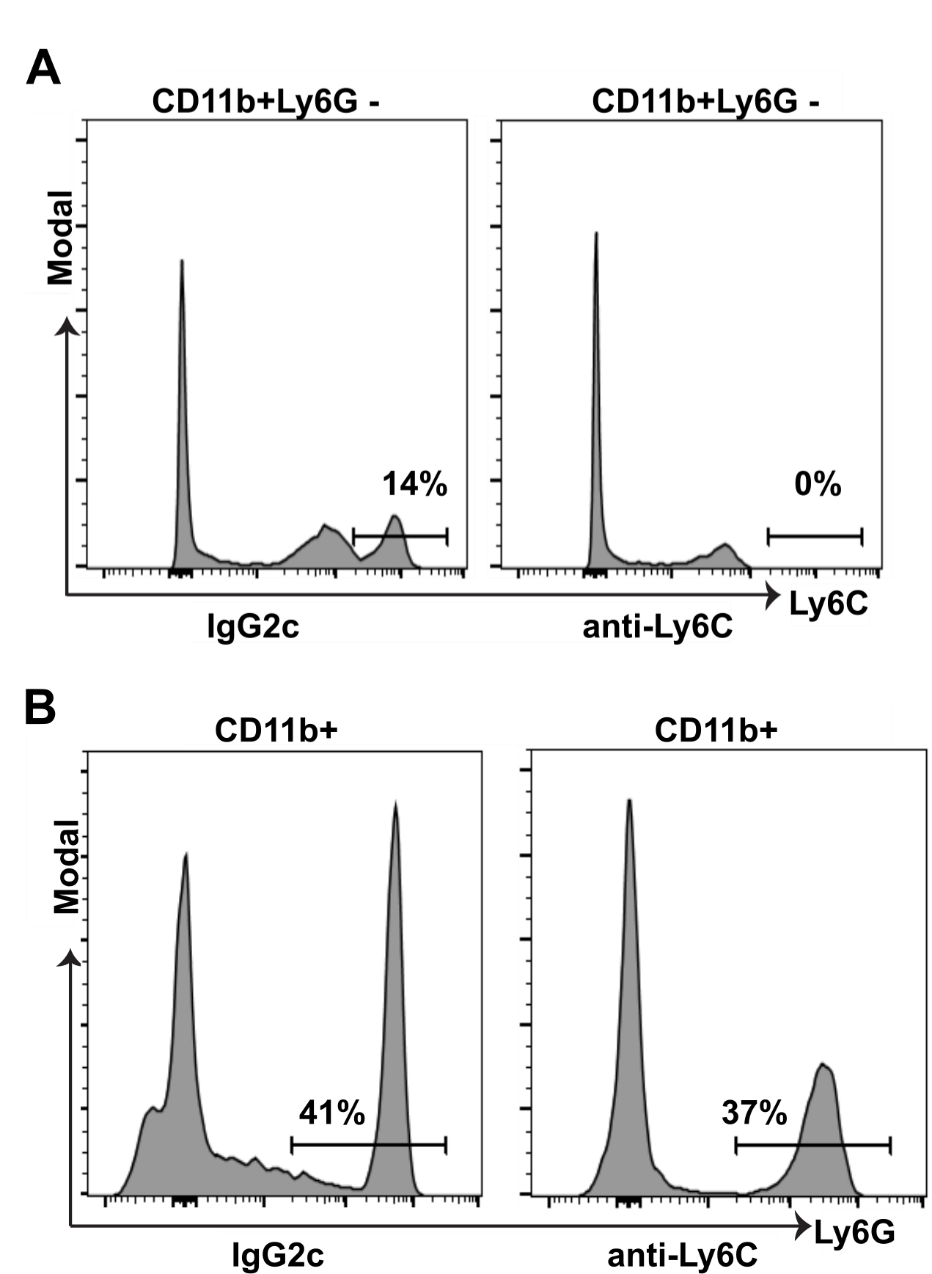


**Figure S3: The anti-Ly6C antibody efficiently depleted classical monocytes but not neutrophils in C57BL/6J mice.**

Efficiency of the monoclonal antibody anti-Ly6C (100 µg per mouse) to specifically deplete classical monocytes in C57BL/6J mice was assessed using flow cytometry. Administration of anti-Ly6C efficiently depleted circulating classical monocytes but did not affect circulating neutrophils. (A) Representative flow cytometric histograms showing CD11b^+^ Ly6G^-^Ly6C^hi^ classical monocytes as a percentage of CD11b^+^ cells; isotype control-treated (left panel), and anti-Ly6C-treated (right panel). (B) Representative flow cytometric histograms showing the percentage of Ly6G^+^ neutrophils on CD11b^+^ cells; isotype control treated (left panel) and anti-Ly6C-treated (right panel).

**Table S1. Macroscopic damage scores**

| **Figure** | **Group** | **DSS Concn.**  **(%)** | **Macroscopic**  **damage score**  **(mean ± SEM)** | **n** | **Significance (p value)** | **t** | **F(DFn, DFd)** | **df** |
| --- | --- | --- | --- | --- | --- | --- | --- | --- |
| 1 | Control |  | 0.4 ± 0.1 | 10 | *** (< 0.001) | 10.6 |  | 18 |
| 1 | Colitis | 2.5 | 4.1 ± 0.3 | 10 |  |  |  |  |
| 2 | Control |  | 0.4 ± 0.2 | 6 | *** (< 0.001) | 8.3 |  | 11 |
| 2 | Colitis | 2.5 | 3.6 ± 0.3 | 7 |  |  |  |  |
| 3A | Colitis + IgG2b | 2.5 | 3.6 ± 0.5 | 8 | ns (0.78) | 0.3 |  | 14 |
| 3A | Colitis + anti-α4 | 2.5 | 3.8 ± 0.6 | 8 |  |  |  |  |
| 3B | Colitis + IgG2a | 2.5 | 4.5 ± 0.6 | 6 | ns (0.22) | 1.3 |  | 12 |
| 3B | Colitis + anti-α4ß7 | 2.5 | 3.3 ± 0.7 | 8 |  |  |  |  |
| 3C | Colitis + IgG2a | 2.5 | 3.8 ± 0.4 | 5 | ns (0.34) | 1 |  | 9 |
| 3C | Colitis + anti-MAdCAM1 | 2.5 | 3.3 ± 0.3 | 6 |  |  |  |  |
| 4 | Control |  | 0.2 ± 0.0 | 6 | ** (< 0.01) | 4.5 |  | 10 |
| 4 | Colitis | 2.5 | 3.5 ± 0.7 | 6 |  |  |  |  |
| 5 | Colitis + IgG2a | 2.5 | 4.2 ± 0.5 | 5 | ns (0.41) | 0.9 |  | 11 |
| 5 | Colitis + anti-Ly6G | 2.5 | 5.0 ± 0.6 | 8 |  |  |  |  |
| 6A | Colitis + IgG2c | 2.5 | 3.8 ± 0.4 | 4 | ns (0.22) | 1.3 |  | 8 |
| 6A | Colitis + anti-Ly6C | 2.5 | 4.7 ± 0.5 | 6 |  |  |  |  |
| 6B | Colitis + IgG2c | 2.5 | 4.0 ± 0.4 | 5 | ns (0.29) | 1.1 |  | 9 |
| 6B | Colitis + anti-Ly6C | 2.5 | 4.7 ± 0.5 | 6 |  |  |  |  |
| 7 | Control + PBS |  | 0.2± 0.0 | 6 | *** (< 0.001) |  | F(2, 15) = 34 |  |
| 7 | Colitis + IgG2a | 3 | 4.3± 0.5 | 6 |  |  |  |  |
| 7 | Colitis + anti-α4ß7 | 3 | 4.4± 0.5 | 6 |  |  |  |  |
| 8 | Control + PBS |  | 1.7 ± 0.3† | 9 | *** (< 0.001) |  | F(3, 29) = 32.4 |  |
| 8 | Control + IL-1RA |  | 2.0 ± 0.4† | 7 |  |  |  |  |
| 8 | DSS + PBS | 3.5 | 6.6 ± 0.4† | 10 |  |  |  |  |
| 8 | DSS + IL-1RA | 3.5 | 5.9 ± 0.7† | 7 |  |  |  |  |
| Add. 6A | Male Control |  | 0.4 ± 0.3 | 4 | *** (< 0.001) | 6.9 |  | 10 |
| Add. 4A | Male Colitis | 2.5 | 4.9 ± 0.4 | 8 |  |  |  |  |
| Add. 6B | Male Control |  | 0.2 ± 0.0 | 4 | *** (< 0.001) | 7.4 |  | 6 |
| Add. 6B | Male Colitis | 2.5 | 7.7 ± 1.0 | 4 |  |  |  |  |
| Add. 11 A, B | Colitis + PBS | 2.5 | 2.3± 0.1 | 5 |  |  |  |  |
| Add. 11 A, B | Colitis + IgG2a | 2.5 | 4.1± 0.4 | 5 | ns (0.15) |  | F (2, 12) = 2.25 |  |
| Add. 11 A, B | Colitis + anti-α4ß7 | 2.5 | 3.1± 0.3 | 5 |  |  |  |  |
| Add. 11 C, D | Colitis + PBS | 2.5 | 4.6± 0.2 | 9 |  |  |  |  |
| Add. 11 C, D | Colitis + IgG2a | 2.5 | 5.6± 0.2 | 9 | P = 0.05‡ |  | F (2, 25) = 3.4 |  |
| Add. 11 C, D | Colitis + anti-MAdCAM-1 | 2.5 | 3.9± 0.1 | 10 |  |  |  |  |

†The scores are higher than typical controls/treated mice likely due to the increased handling stress associated with the ICV cannulation. Control animals lost weight, but had no appreciable colonic damage, and the weight loss in colitic mice was greater than normal. The other parameters of the macroscopic damage in the colitic groups were similar to that of all other treated groups.

‡ Anti-MAdCAM-1 treated mice with colitis had a significantly (P=0.04) lower damage score than mice treated with colitis treated with IgG2a, but neither group were significantly different from those treated with PBS.


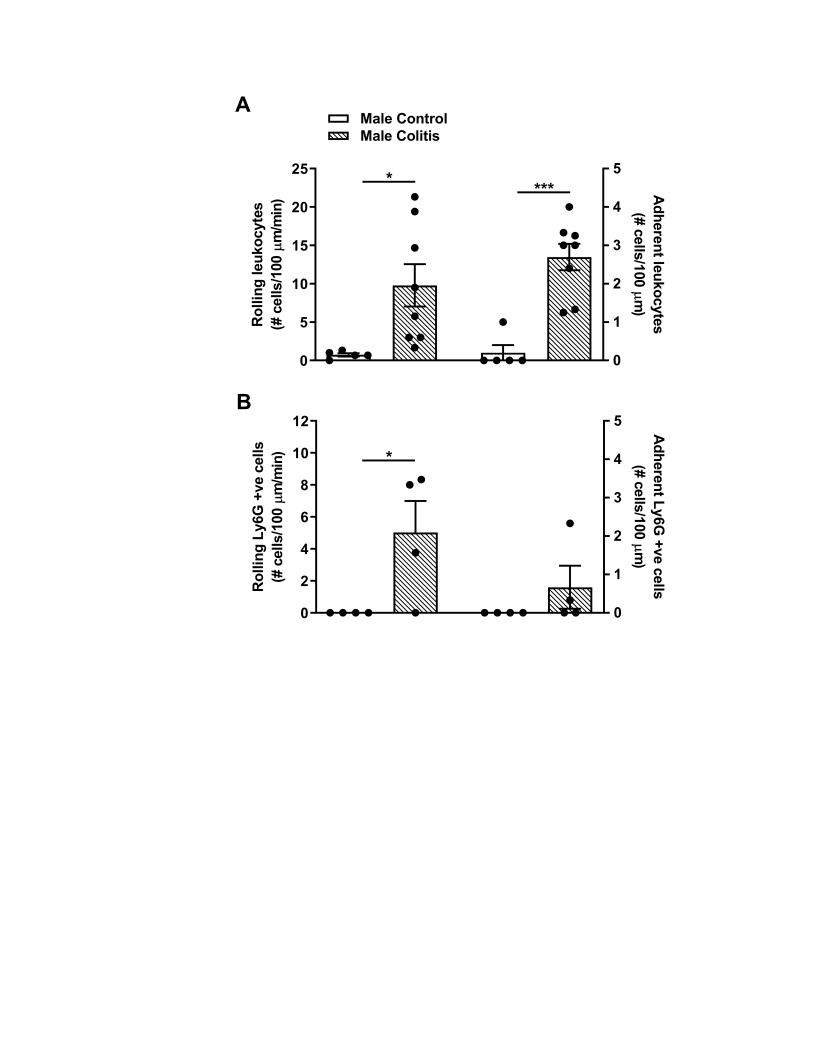


**Figure S4: Colitis induces the rolling and adherence of leukocytes and the rolling of neutrophils along cerebral endothelial cells of male mice.**

Intravital microscopy was performed using a spinning disc confocal microscope. Videos were captured and analyzed to identify rolling and adhering of leukocytes in control and colitic mice. (A) Colitic male mice showed a significant increase in the rolling (t=2.3, df 10, *p=0.047, n=5-8 mice/group) and adhering (t=4.6, df 10, ***p<0.001, n=4-8 mice/group) of leukocytes in CECs. (B) Colitis significantly increases the rolling (t=2.5, df 6, *p=0.044; n=4 mice/group) but not adhering (t=1.2, df 6, p<0.28; n=4 mice/group) of neutrophils (Ly6G positive cells) in colitic male mice compared to controls.

**Table S2. Supplementary cytokine data**

| **Cytokine** | **Group** | **Average concentration in pg/mg**  **(mean ± S.E. mean)** | **n** | **Significance**  **(p value)** | **F(DFn, DFd)** |
| --- | --- | --- | --- | --- | --- |
| GM-CSF | Control + PBS | 0.1 ± 0.0 | 6 | ns (0.15) | F(2, 14) = 2.2 |
|  | Colitis + IgG2a | 0.2 ± 0.0 | 6 |  |  |
|  | Colitis + anti-α4ß7 | 0.1 ± 0.0 | 5 |  |  |
| IL-2 | Control + PBS | 0.2 ± 0.0 | 6 | * (0.02) | F(2, 15) = 5.2 |
|  | Colitis + IgG2a | 0.4 ± 0.1 | 6 |  |  |
|  | Colitis + anti-α4ß7 | 0.3 ± 0.1 | 6 |  |  |
| IL-6 | Control + PBS | 0.0 ± 0.0 | 5 | * (0.01) | F(2, 14) = 6.4 |
|  | Colitis + IgG2a | 0.1 ± 0.0 | 6 |  |  |
|  | Colitis + anti-α4ß7 | 0.1 ± 0.0 | 6 |  |  |
| IL-12p70 | Control + PBS | 0.1 ± 0.0 | 6 | ns (0.06) | F(2, 12) = 3.6 |
|  | Colitis + IgG2a | 0.3 ± 0.1 | 6 |  |  |
|  | Colitis + anti-α4ß7 | 0.3 ± 0.1 | 3 |  |  |

Samples analyzed for IL-4, IFNγ and TNF were below the level of detection.

**
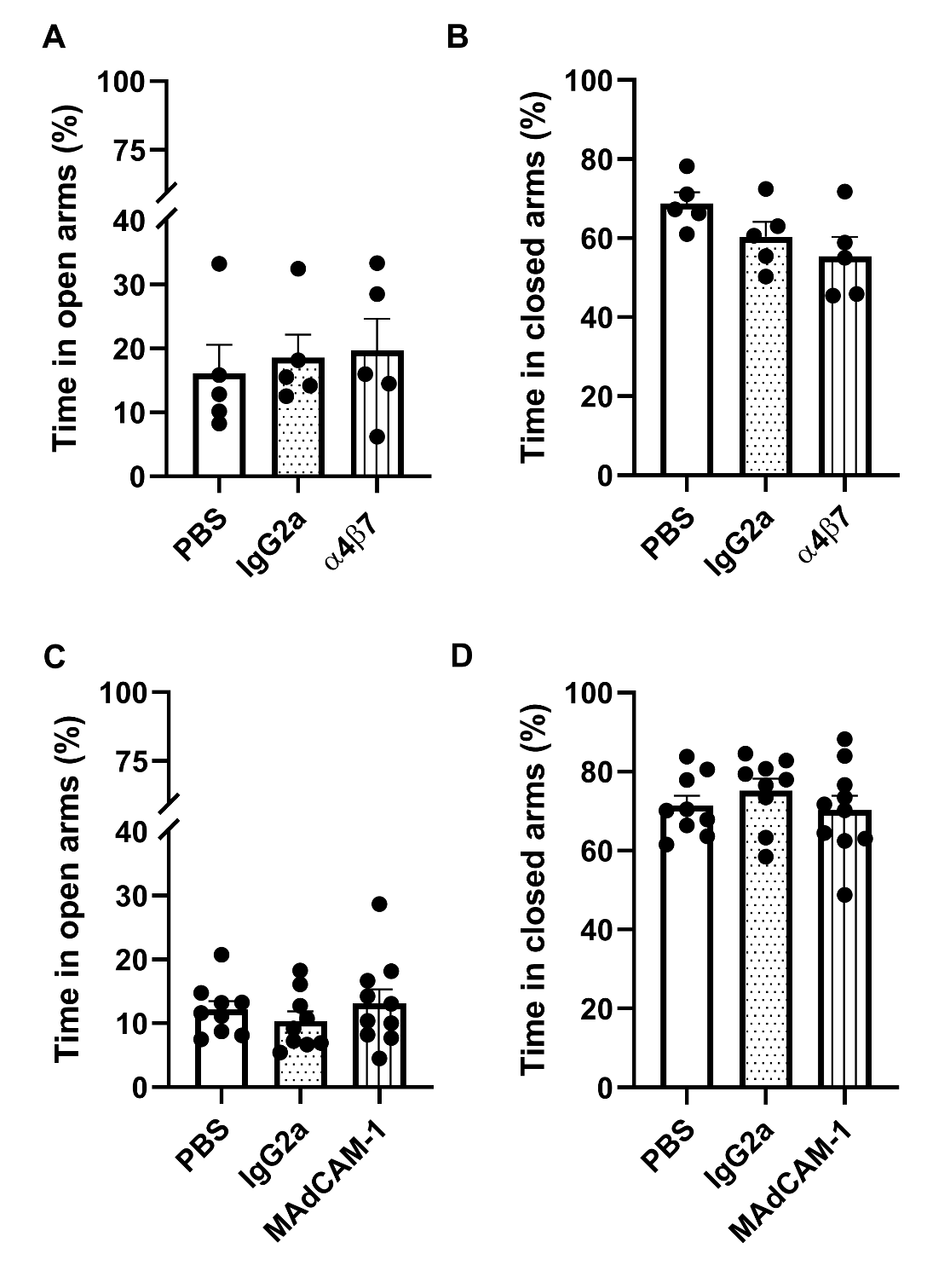
**

**Figure S5. In vivo neutralization of α4β7 integrin or anti-MAdCAM-1 does not reduce anxiety-like phenotype in colitic mice.** To investigate the effect of blocking α4β7 integrin or MAdCAM-1 on behavior, colitic female mice were assessed after anti-α4β7 or anti-MAdCAM-1 treatment mice. On day 4 and 6 of DSS treatment, the control group (n=5) was administered sterile phosphate-buffered saline (PBS) 10mL/kg, i.p. while the DSS-treated mice were given either control IgG2a antibody (200 μg/mouse, IP; Bio X Cell; catalog #BE0089, n=5), or anti-α4β7 integrin antibody (200 μg/mouse, IP; Bio X Cell; catalog #BE0034, n = 5) or on days 3 and 5 of DSS treatment, other mice were (n=9) were administered sterile phosphate-buffered saline (PBS) 10mL/kg, i.p. while the DSS-treated mice were given either control IgG2a antibody (200 μg/mouse, IP; Bio X Cell; catalog #BE0089, n=10), or anti-MAdCAM-1 (MECA-367; 200 μg/mouse; Bio X Cell; catalog #BE0035, n=10) to investigate the role of integrins in behavioral changes. At peak colitis, mice were assessed for anxiety-like behavior using the elevated plus maze. **A.** In colitic mice, anti-α4β7 did not significantly alter the percentage time spent in the open arms of the maze (F2, 12) = 0.18, P=0.84; one-way ANOVA). **B.** Similarly, anti-α4β7 did not significantly alter the percentage time spent in the closed arms of the maze (F(2, 12) = 3.0, P=0.09; one-way ANOVA). **C.** In colitic mice, anti-MAdCAM-1 did not significantly alter the percentage time spent in the open arms of the maze (F2, 25) = 0.64, P=0.53; one-way ANOVA). **D.** Similarly, anti-MAdCAM-1 did not significantly alter the percentage time spent in the closed arms of the maze (F(2, 25) = 0.69, P=0.51; one-way ANOVA).
